# Beneficial Effects of *Sideritis clandestina* Extracts and Sideridiol against Amyloid β Toxicity

**DOI:** 10.3390/antiox13030261

**Published:** 2024-02-21

**Authors:** Anna Gioran, Yiorgos Paikopoulos, Eleni Panagiotidou, Aikaterini E. I. Rizou, Georgia I. Nasi, Virginia D. Dimaki, Konstantina D. Vraila, Dimitra S. Bezantakou, Panagiotis M. Spatharas, Nikos C. Papandreou, Vassiliki Magafa, Fotini N. Lamari, Vassiliki A. Iconomidou, Niki Chondrogianni

**Affiliations:** 1 Institute of Chemical Biology, National Hellenic Research Foundation, 48 Vassileos Constantinou Avenue, 11635 Athens, Greece; agioran@eie.gr (A.G.); yiorgos.paikopoulos@isas.de (Y.P.); epanagiotidou@eie.gr (E.P.); 2 Department of Biochemistry and Biotechnology, University of Thessaly, 41334 Larissa, Greece; 3 Section of Cell Biology and Biophysics, Department of Biology, School of Sciences, National and Kapodistrian University of Athens, Panepistimiopolis, 15701 Athens, Greece; rizoukat@biol.uoa.gr (A.E.I.R.); gnasi@biol.uoa.gr (G.I.N.); vrailakon@biol.uoa.gr (K.D.V.); dimbez@biol.uoa.gr (D.S.B.); panspatharas@biol.uoa.gr (P.M.S.); npapand@biol.uoa.gr (N.C.P.); veconom@biol.uoa.gr (V.A.I.); 4 Laboratory of Pharmacognosy and Chemistry of Natural Products, Department of Pharmacy, University of Patras, 26504 Patras, Greece; virnadimaki@upatras.gr (V.D.D.); magafa@upatras.gr (V.M.); flam@upatras.gr (F.N.L.)

**Keywords:** *Sideritis clandestina* spp. *peloponnesiaca*, Alzheimer’s disease, *Caenorhabditis elegans*, amyloid beta peptide, protein aggregation, sideridiol, verbascoside, natural antioxidant products

## Abstract

Alzheimer’s disease (AD) is the most common form of dementia. Given the link between oxidative stress and AD, many studies focus on the identification of natural antioxidants against AD. Although their antioxidant capacity is important, increasing data suggest that additional activities are related to their beneficial effects, including properties against amyloid beta (Aβ) aggregation. *Sideritis* spp. (mountain tea) extracts possess not only antioxidant activity but also other bioactivities that confer neuroprotection. Although various *Sideritis* spp. extracts have been extensively studied, there are scarce data on *S. clandestina* subsp. *peloponnesiaca* (SCP) phytochemical composition and neuroprotective potential, while nothing is known of the responsible compounds. Given that SCP is a weaker antioxidant compared to other *Sideritis* spp., here, we investigated its potential beneficial properties against Aβ aggregation. We characterized different SCP extracts and revealed their anti-aggregation activity by taking advantage of established *C. elegans* AD models. Importantly, we identified two pure compounds, namely, sideridiol and verbascoside, being responsible for the beneficial effects. Furthermore, we have revealed a potential anti-Aβ aggregation mechanism for sideridiol. Our results support the use of mountain tea in the elderly against dementia and demonstrate the activity of sideridiol against Aβ aggregation that could be exploited for drug development.

## 1. Introduction

Alzheimer’s disease (AD) is the most common form of dementia, affecting millions of people either directly as patients or indirectly as carers and family members. It is a severe, progressive neurodegenerative disease without an effective cure. The currently used drugs or disease-modifying therapies mainly reduce the disease symptoms. Moreover, the approved drugs exhibit short-term benefits accompanied by various side effects [1]. Given that aging (and the accompanying oxidative burden) represents a major risk factor for AD, the problem is further aggravated by the ever-growing aged human population [2]. With an exponentially increasing number of elderly people in Western societies, we urgently need to identify strategies to prevent or delay age-associated frailty and diseases to extend healthspan and prolong lifespan. To decrease the possibility of negative side effects that usually emerge with current medications, a lot of research effort is put into the identification of new agents to prevent, decelerate, or even treat AD. Natural products are privileged candidates due to their potential for pleiotropic neuroprotective activities, including antioxidant and/or anti-inflammatory activities and inhibitory activity against production and aggregation of amyloid beta (Aβ) [3,4,5]. Indeed, medicinal plants have been the main source of drug discovery, e.g., galanthamine from *Galanthus nivalis* L. [6,7], but their study is far from complete since the number of plant taxa is huge, and the nature and the properties of their secondary metabolites have only partially been explored.

On the Balkan Peninsula, *Sideritis* species (mountain tea, ironwort), well-known for their antioxidant properties [8], are consumed daily as decoctions/infusions for their exquisite taste and aroma but are also traditionally used to prevent age-related problems in the elderly, including gastrointestinal and respiratory diseases, the common cold as well as a diuretic [9,10]. There are more than 150 species in the genus *Sideritis* (Lamiaceae family) [11]. They are primarily distributed throughout the Balkan Peninsula and Mediterranean region [12]. Various secondary metabolites belonging to different chemical families such as flavonoids, terpenoids, iridoids, sterols, coumarins, and lignans, have been identified in *Sideritis* taxa [11]. The genus is represented by 16 taxa in Greece, five of which are geographically endemic to certain mountainous regions of the country. The monograph published by EMA (EMA/HMPC/39453/2015) [13], which includes *Sideritis scardica* Griseb., *S. clandestina* (Bory and Chaub.) Hayek, *S. raeseri* (Boiss. and Heldr.), and *S. syriaca* L., reflects the usage of all taxa under the same name and for the same purpose. *S. clandestina* is a variable hemicryptophytic species, endemic to the Greek mountains of Peloponnese, found at altitudes from 1600 m to 2300 m. Taxonomically, two subspecies can be recognized: *S. clandestina* (Bory and Chaub.) Hayek subsp. *peloponnesiaca* (Boiss. and Heldr.) Baden (SCP), endemic to Central and Northern Peloponnese mountains; and *S. clandestina* (Bory and Chaub.) Hayek subsp. *clandestina* (SCC), mainly found on Taygetos and Parnon (southern mountains [14]).

The endemic species, SCP and SCC, have not been largely studied. In addition to studies on their essential oils, we have previously isolated and characterized nine polar metabolites (iridoid glycosides, phenolic acid glycosides, caffeoyl ester glycosides) from SCP and we showed its distinct phytochemical differences from three other *Sideritis* taxa (SCC, *S. raeseri* and *S. scardica*) in petroleum ether and aqueous extracts [15]. Current metabolomic approaches demonstrate that the phytochemical composition of medicinal plants, in general, and of *Sideritis* in particular, varies among the taxa and depends on the genotype (thus explaining differences even among the same taxon), the environmental conditions, the time of collection, the plant part, and the extraction process [16,17]. This inherent natural product richness and chemovariability necessitates the strict quality control of extracts and impacts greatly on their bioactivity.

Oxidative stress is a major AD hallmark that exacerbates AD progression [18]. Various studies have suggested the lower probability of AD onset and progression upon consumption of antioxidants [19,20,21]. Consequently, the therapeutic potential of natural antioxidants against AD has been increasingly investigated [22]. Under these terms, the ability of the mountain tea to prevent or reverse the AD phenotype has been investigated for various *Sideritis* spp. (mainly other than SCP) in various cellular and animal models. Notably, additional mechanisms of action of the *Sideritis* spp. extracts have been revealed. More specifically, *S. scardica* extracts have been shown to act as inhibitors of the uptake of some neurotransmitters involved in various neurological disorders [23] or to modulate the AMPA receptor-dependent neurotransmission [24]. They were also shown to promote non-amyloidogenic amyloid precursor protein (APP) processing and to reduce total and hyperphosphorylated tau protein levels in cellular models for AD [25]. Similarly, various fractions of *S. scardica* extract demonstrated activity against Aβ toxicity, while antioxidant properties were revealed for almost all these fractions [26]. The mid-polar extracts of *S. scardica* conferred a reduction in Aβ aggregates in various AD *Caenorhabditis elegans* models, accompanied by a significantly delayed paralysis rate [27]. Treatment of aged, non-transgenic, and APP-transgenic mice with *S. scardica* extract (and *S. euboea* extract) enhanced cognition and reduced the amyloid plaque burden in transgenic mice [28]. Nevertheless, results were not equally promising when female mice were used while high doses induced a dose-dependent sedative effect [29]. More recently, *S. scardica* water extract was demonstrated to preserve memory in mice with scopolamine-induced dementia [30]. The only study with SCP showed that consumption of 4% herbal tea for 6 weeks increased the total antioxidant capacity of specific cerebral regions of adult male mice [31], whereas the same scheme with SCC herbal tea induced anxiolytic-like effects [32]. It is, however, noteworthy that although antioxidant protection was demonstrated by SCP [31], SCP has a lower phenolic content and weaker antioxidant properties than other *Sideritis* spp., including *S. scardica* [15]. Consequently, there are hints that any beneficial effects of SCP might occur via additional pathways on top of its antioxidant properties.

Human studies have been conducted with *S. scardica* extract. More specifically, a double-blind, randomized, placebo-controlled, parallel groups study in healthy volunteers following acute (1 day) and a 28 days-long consumption period of *S. scardica* extract revealed significant improvement of cognitive performance at both time points [33]. Moreover, stress-induced impairment of executive functioning was alleviated in young individuals upon dietary supplementation of an *S. scardica* extract and selected B-vitamins [34]. Finally, in a double-blind, randomized, and placebo-controlled clinical trial, a combination of *S. scardica* and *Bacopa monnieri* extract positively affected the mental performance of subjects suffering from mild cognitive impairment (MCI), a transitional stage during AD progression [35].

Despite the above-mentioned neuroprotective properties of *S. scardica* and (much less of) *S. clandestina* extracts, it is noteworthy that in most cases, the extracts were not characterized, and pure distinctive natural products have not been investigated. Moreover, very scarce data exist on SCP and its neuroprotective potential on top of its antioxidant capacity. We therefore investigated the potential effects of SCP against Aβ toxicity by taking advantage of established *C. elegans* strains widely used as AD models [36]. We studied the effects of the different SCP extracts (characterized with LC-MS) and their most abundant ingredients. We are the first to reveal their protective effects against Aβ aggregation and to identify at least two of their pure compounds being, at least partially, responsible for the beneficial outcomes. Our results support the traditional use of mountain tea in the elderly against dementia and give novel insights into the responsible bioactive compounds that could serve as lead compounds for drug development.

## 2. Materials and Methods

### 2.1. Plant Material and Preparation of Extracts and Compounds

#### 2.1.1. Plant Material and Preparation of Extracts for Analysis and Characterization

Aerial SCP parts were collected from the mountain of Chelmos (Kalavrita, Greece) in June and authenticated by Prof. Gregorios Iatrou (Department of Biology, University of Patras). Plant specimens were deposited at the Herbarium of the University of Patras with a voucher number (UPA22921). The dried SCP aerial parts (943 g) were successfully extracted with solvents of increasing polarity: 10 L of petroleum ether, 7.5 L of ethyl acetate (EtOAc), and 7 L of methanol (MeOH), as earlier described [15]. The volumes are the total volumes of the extractants since the plant was extracted five times with each solvent at room temperature (one day every time). The solvents were evaporated in vacuo, and a quantity of the dried extracts was re-dissolved and processed with activated charcoal to remove chlorophylls, filtered, and dried again in a rotary evaporator. Those were used for bioassays and the LC-MS analysis.

#### 2.1.2. Preparation of Extracts and Compounds for *C. elegans* Treatment

SCP (MeOH), SCP (EtOAc), siderol, and sideridiol were resuspended in DMSO at 10 mg/mL for sideridiol and siderol and 20 mg/mL for the rest. Verbascoside and chlorogenic acid were resuspended in H_2_O at 20 and 10 mg/mL, respectively. Dilutions of the stock solutions to reach the working concentrations indicated throughout the manuscript were performed with M9 buffer before added on plates seeded with UV-inactivated OP50.

#### 2.1.3. Dissolution of Sideridiol and Sample Preparation for TEM Experiments

Sideridiol was dissolved in DMSO at a concentration of 10 mg/mL. The Aβ_42_ peptide-containing films (see below) were left at room temperature for 30 min and then mixed with the solution of sideridiol in the following ratios (Aβ_42_:sideridiol): 1:1, 1:5, and 1:10. Individual Aβ_42_ solution served as control in all in vitro experimental assays. Depending on the assay, the peptide concentration was set to 10 μM or 40 μM, and the DMSO concentration was kept below 3% to prevent changes in Aβ_42_ aggregation kinetics. Each sample was exposed to a 60 s 35 °C ultrasound water bath. Following this, 0.1 M HEPES, pH 7.5, was added to reach the desired final volume, and the samples were agitated at 37 °C for two hours and then incubated at 37 °C for a minimum of seven days.

### 2.2. LC-MS Analysis of the Extracts

The single quadrupole LC/MS system of LC/MSD1260 Infinity II (Agilent Technologies, Inc., Santa Clara, CA, USA) was used. The system was equipped with an ESI ion source and the mass range of the single quadrupole mass analyzer was *m*/*z* 100–1600 in full scan mode. Nitrogen was used as the gas for ionization. Working conditions were in ESI negative mode and separation was performed on a reversed phase Poroshell 120 EC-C-18 column (120 Å, 2.7 μm, 4.6 × 150 mm) (Agilent Technologies, Inc., Santa Clara, CA, USA).

For SCP (EtOAc) extract analysis, the mobile phase consisted of H_2_O-0.1% CH_3_COOH (solvent A) and CH_3_CN-0.1% CH_3_COOH (solvent B). The flow rate was 0.5 mL/min, the temperature of the column was 25 °C, and the gradient was as follows: 40% B (0–2 min); 40–65% B (2–3 min); 65% B (3–38 min); 65–90% B (38–55 min); 90% B (55–65 min); 90–40% B (65–70 min). The sample concentration was 0.5 mg/mL. Sideridiol (isolated by our group, see below) was used as an external standard in concentrations ranging from 0.002 to 0.1 mg/mL. The calibration curve was *y* = 2 × 10^8^*x* + 1 × 10^6^ (*R*^2^ = 0.9896).

For SCP (MeOH) extract analysis, the mobile phase consisted of 0.1% acetic acid in water (A) and in MeOH (B) as previously described [15]. Briefly, separation was carried out in 65 min under the following conditions: 15% B (0–8 min); 15–35% B (8–13 min); 35% B (13–18 min); 35–40% B (18–19 min); 40% B (19–27 min); 40–45% B (27–28 min); 45% B (28–35 min); 45–75% B (35–45 min); 75% B (45–55 min); 75–15% B (55–59 min); 15% B (59–65 min). The flow rate was 0.3 mL/min. The samples were prepared by diluting the dry extracts in MeOH-1% CH_3_COOH to a final concentration of 1 mg/mL. Quantification was performed using rutin (HPLC > 99%) from Extrasynthese (Genay, France) as an external standard.

### 2.3. Isolation of the Most Abundant Natural Products in the Two Extracts

The SCP (EtOAc) extract was concentrated in vacuo, yielding 13.01 g, and then fractionated on a silica gel column chromatography (CC1) with solvents of increasing polarities, i.e., hexane, EtOAc, acetone, MeOH, and finally, MeOH: H_2_O (70:30) with 1% acetic acid, to obtain five fractions (Fr.A-Fr.E). Fr.B (5.56 g) was submitted to silica gel CC2 with dichloromethane (DCM), dichloromethane–acetone (90:10, 80:20, 50:50, and 10:90), acetone–MeOH (90:10), affording ten fractions (Fr.B1-Fr.B10). To remove chlorophylls, Fr.B3 (0.38 g), Fr.B4 (0.50 g), Fr.B5 (0.49 g), and Fr.B7 (0.41 g) were further treated with activated charcoal, resulting in Fr.B3′ (0.24 g), Fr.B4′ (0.40 g), Fr.B5′ (0.22 g), and Fr.B7′ (0.39 g).

Fr.B4′ (0.15 g), after crystallization with MeOH, was subjected to silica gel CC3 with hexane–DCM (from 100:0 to 60:40), hexane–DCM (60:40) + 1% MeOH *v*/*v*, hexane–DCM (60:40) + 5% MeOH *v*/*v*, and hexane–DCM (50:50) + 5% MeOH *v*/*v* to give eleven fractions (Fr.B4′.1-Fr.B4′.11). Fr.B4′.6 afforded siderol (64 mg).

Fr.B7′ was crystallized with EtOAc and the precipitate (0.075 g) was further purified with PTLC (silica gel 60, 20 × 20 cm, 0.5 mm). A total of 0.058 g was absorbed on three plates (silica gel 60, 20 × 20 cm, 0.5 mm) and each plate was eluted four times with hexane–EtOAc (60:40, *v*/*v*). The fraction with Rf = 0.45 afforded sideridiol (18.6 mg).

**Figure 1:**
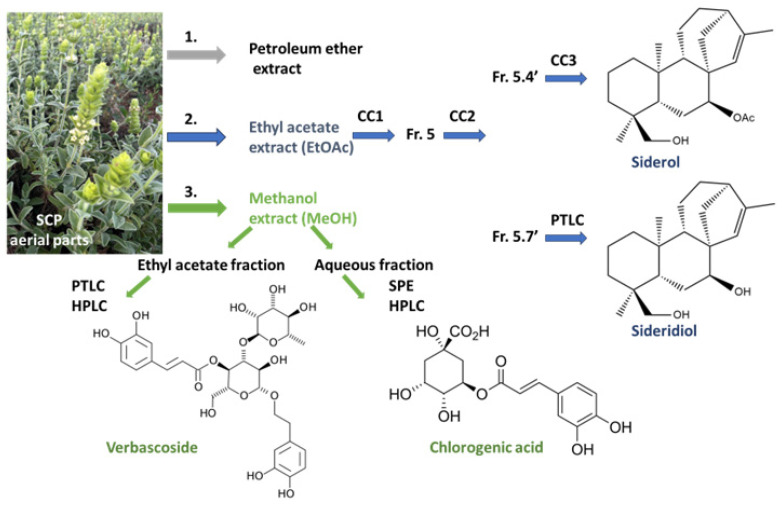
Outline of the isolation procedures of the *Sideritis clandestina* spp. *peloponnesiaca* (SCP) ethyl acetate (EtOAc) and methanol (MeOH) extracts and the four major natural products (siderol, sideridiol, verbascoside, chlorogenic acid) that were tested in this study. SCP aerial parts were subsequently extracted with 1. petroleum ether; 2. EtOAc (EtOAc extract); and 3. MeOH (MeOH extract). After multiple column chromatography (CC) steps of the original EtOAc extract and the subsequent fractions (Fr.5, Fr.5.4′) and preparative thin-layer chromatography (PTLC) of Fr.5.7′, the two ent-15-kaurene diterpenes, siderol and sideridiol, were isolated. The MeOH extract was partitioned with different solvents yielding the EtOAc and the aqueous fractions. Verbascoside was isolated from the former EtOAc fraction after PTLC and preparative HPLC, whereas chlorogenic acid was isolated from the aqueous fraction after solid phase extraction (SPE) and preparative HPLC.

The SCP (MeOH) extract, verbascoside [β-(3′,4′-dihydroxyphenyl)ethyl-*O*-α-l-rhamnopyranosyl(1→3)-β-d-(4-*O*-caffeoyl)-glucopyranoside] and chlorogenic acid (5-*O*-caffeoyl quinic acid) were isolated and identified, as previously described [15]. Figure 1 shows a brief outline of the isolation procedures along with the structures of the main natural products investigated in the current study.

### 2.4. Characterization of the Natural Products

The purity and the molecular weights of both isolated compounds were determined on the single quadrupole LC/MS system of LC/MSD1260 Infinity II (Agilent Technologies, Inc., Santa Clara, CA, USA), and the NMR spectra were recorded on a Bruker 700 MHz Avance III HD Ascend TM spectrometer.

Siderol (ent-7-acetyl-18-hydroxykaur-15-ene): mp: 146–147 °C.

1H NMR (700 MHz, Chloroform-d) δ 5.26 (s, 1H), 4.70 (dd, J = 3.6, 2.2 Hz, 1H), 3.33 (d, J = 10.8 Hz, 1H), 3.00 (d, J = 10.8 Hz, 1H), 2.38–2.36 (m, 1H), 2.07 (s, 3H), 1.96 (d, J = 10.0 Hz, 1H), 1.84–1.78 (m, 1H), 1.75 (ddd, J = 14.4, 3.7, 1.7 Hz, 1H), 1.70 (d, J = 1.6 Hz, 3H), 1.66–1.64 (m, 1H), 1.54–1.45 (m, 9H), 1.36 (t, J = 4.0 Hz, 1H), 1.25–1.22 (m, 1H), 1.08 (s, 3H), 0.84 (td, J = 13.0, 3.6 Hz, 1H), 0.71 (s, 3H).

13C NMR (176 MHz, Chloroform-d) δ 170.89, 143.94, 129.94, 78.40, 71.55, 52.01, 45.01, 44.70, 42.15, 40.00, 39.95, 39.24, 37.14, 35.36, 24.93, 23.65, 21.61, 18.45, 18.08, 17.91, 17.51, 15.52.

UHPLC-MS: *m*/*z* = 369 [M + Na]^+^/715 [2M + Na]^+^. The molecular weight calculated for C_22_H_34_O_3_ was 346.25. UHPLC-MS purity: 94%.

IR: vmax 3469 (OH), 2921 (C¬H), 1707 (C=O), 1263 (C¬O) cm^−1^.

The spectroscopic data agree with previous studies describing its characterization in other *Sideritis* species [37,38,39].

Sideridiol (ent-7,18-dihydroxykaur-15-ene): mp: 193 °C.

1H NMR (700 MHz, MeOH-d) δ 5.54 (s, 1H), 3.55 (t, 1H, J = 2.8 Hz), 3.32 (d, 1H, J = 11.0 Hz), 3.08 (d, 1H, J = 11.2 Hz), 2.36–2.35 (m, 1H), 2.00 (d, 1H, J = 9.8 Hz), 1.83 (dd, 1H, J = 2.6, 12.6 Hz), 1.73 (d, 3H, J = 1.4 Hz), 1.73–1.71 (m, 1H), 1.61–1.59 (m, 2H), 1.58–1.55 (m 5H), 1.54–1.53 (m, 2H), 1.49–1.47 (m, 2H), 1.44 (dd, 1H, J = 4.2, 13.3 Hz), 1.38 (m 1H), 1.36–1.34 (m, 1H), 1.33–1.31 (m, 1H,), 1.12 (s, 3H), 0.84 (td, 1H, J = 3.5, 12.6 Hz), 0.78 (s, 1H). 

13C NMR (176 MHz, MeOH-d) δ 144.10, 131.96, 76.29, 72.36, 54.57, 46.07, 45.21, 43.49, 41.20, 40.99, 40.53, 38.25, 36.55, 27.60, 25.96, 19.47, 19.07, 18.56, 17.65, 15.5. 

UHPLC-MS: *m*/*z* = 327 [M + Na]^+^/631 [2M + Na]^+^. The molecular weight calculated for C_20_H_32_O_2_ was 304.47; UHPLC-MS purity: 93%.

IR: v_max_ 3541 (OH), 3443 (OH), 3058 (=C¬H), 1657 (=C¬H) cm^−1^.

The spectroscopic data are in unison with previous studies describing its characterization in other *Sideritis* species [37,40].

### 2.5. C. elegans Strains and Culture

Standard techniques for the maintenance of all nematode strains were followed [41], with animals grown on OP50 (a bacterial strain conventionally used as food for laboratory maintenance of *C. elegans*) and maintained at 20 °C unless otherwise indicated. The following *C. elegans* strains were obtained from the Caenorhabditis Genetics Center (CGC): wild type (wt) N2 (Bristol); CL4176 dvIs27[myo-3p::Aβ_1–42_::let-851 3′UTR + rol-6(su1006)]); GMC101 dvIs100[unc-54p::Aβ_1–42_::unc-54 UTR + mtl-2p::GFP]; CL2331: dvIs37 [myo-3p::GFP::Aβ_3–42_ + rol-6(su1006)].

### 2.6. Paralysis Assays

For CL4176 strain, 150–300 animals of L3 stage (already on plates seeded with UV-inactivated OP50 and each extract/compound from the egg stage) were shifted from 16 °C to 25 °C, and paralysis scoring was initiated after 24 h until the entire population was paralyzed (after ~10–14 h). For GMC101 strain: at least 120 animals of L4 stage were transferred on plates with nematode growth medium (NGM) with or without the extract or compound to be tested, seeded with UV-inactivated OP50. When the animals reached the young adult stage, they were shifted from 20 °C to 25 °C. Paralysis scoring was initiated 24 h after temperature upshift until all animals were paralyzed. Animals were scored as paralyzed if they failed to undergo full body wave propagation upon prodding, and each paralysis assay was repeated at least three times (unless otherwise indicated).

### 2.7. Microscopy of CL2331 Animals and Image Analysis

For Aβ_3–42_ deposit measurements, synchronized CL2331 animals were exposed to each examined extract/compound or the diluent (DMSO or H_2_O depending on the extract/compound) and grown at 20 °C to induce the aggregation of the Aβ_3–42_ peptide on plates seeded with UV-inactivated OP50 until day 1 of adulthood. Animals were mounted on 2% agarose pads on glass slides, anesthetized with 25 mM levamisole (Sigma-Aldrich, St. Louis, MO, USA), dissolved in M9 buffer, and observed at room temperature using a Leica TSC SPE confocal laser scanning microscope (Leica Lasertechnik GmbH, Heidelberg, Germany). The LAS AF software was used for image acquisition. Images focused in the anterior area of the nematodes were acquired with a 20/0.70 objective, and Z-stacks were obtained with stable laser intensity and gain throughout all conditions of every experiment. Counting of the number of aggregates was performed in at least 25 animals per condition.

### 2.8. Phenotypic Analysis

N2 animals laid eggs for 20–30 min on NGM plates containing either the tested extract or compound or the diluent (DMSO or H_2_O). The following phenotypic characteristics were monitored as previously described [42,43].

Developmental timing: The offspring were frequently observed to record the time needed to reach the L4 larval stage from egg hatching. The experiment was repeated at least three times.

Fecundity assay/Egg lethality: Single N2 L4 larvae were transferred on NGM plates containing either each extract/compound or the diluent (DMSO or H_2_O). Each animal was transferred every day to a fresh NGM plate containing each extract/compound or the relative diluent. The number of offspring of each animal was scored when they had reached the L2–L3 larval stage. To monitor egg lethality, non-hatched eggs were also scored. At least 5 animals per condition were examined.

Pharyngeal pumping: The pharyngeal pumping rate was measured at day 1 of adulthood. At least 35 animals per condition were examined.

Defecation assay: The defecation rate, that is, the time needed in seconds from one defecation to the next, was measured at day 1 of adulthood. At least 30 animals per condition were examined.

### 2.9. Protein Extraction and Dot Blotting

RIPA buffer supplemented with proteinase inhibitors was used for the lysis of nematodes via sonication. Protein concentration was determined with DC assay (Bio-Rad, Hercules, CA, USA), and 15–20 μg of total protein extract was separated with SDS-PAGE or 5–7 μg of total protein extract was directly loaded on a nitrocellulose membrane mounted on a dot blotter. Coomassie brilliant blue staining was used for equal loading control. The anti-6E10 (BioLegend, San Diego, CA, USA) antibody and the anti-mouse (Santa Cruz, TX, USA) secondary antibody were used.

### 2.10. Synthesis and Disaggregation of Aβ_42_ Peptide

Aβ_42_ peptide (DAEFRHDSGYEVHHQKLVFFAEDVGSNKGAIIGLMVGGVVIA) was synthesized and lyophilized by GeneCust© (Boynes, France) with a >95% purity and both N- and C-terminals being free. The peptide was dissolved in 1,1,1,3,3,3-Hexafluoro-2-propanol (HFIP) (Sigma-Aldrich, St. Louis, MO, USA) at a concentration of 1 mg/mL. The peptide solution was then divided into desired volumes and left to dry overnight in a fume hood at room temperature, thus forming peptide-containing films that were stored at −20 °C for further use.

### 2.11. Transmission Electron Microscopy (TEM) and Negative Staining

TEM was used to examine the potential inhibition of Aβ_42_ fibrillization by sideridiol. More specifically, 10 μL of each 40 μM sample was placed on glow-discharged 400-mesh carbon-coated copper grids and allowed to adhere for 20 min. The grids were then placed on a droplet of 2% (*w*/*v*) aqueous uranyl acetate for 50 s and rinsed three times with distilled water drops to remove excess stain. Whatman filter paper was used to remove excess water and the grids were air-dried. Sample observation was performed in a MorgagniTM 268 Transmission Electron Microscope (FEI, Hillsboro, OR, USA), operated at 80 kV. Digital acquisitions were collected with an 11 Mpixel side-mounted Morada CCD camera (Soft Imaging System, Muenster, Germany), and ImageJ was used for image analysis of the electron micrographs [44].

### 2.12. Thioflavin T (ThT) Kinetic Assay

Measurements of ThT fluorescence were carried out at 37 °C in black 96-well plates with flat, clear bottom on a Tecan Spark microplate reader (Tecan, Mannedorf, Switzerland). The Aβ_42_ peptide-containing films were initially dissolved in DMSO, and the solution of sideridiol was then added at the respective ratios (1:1, 1:5, 1:10); HEPES was added to reach the desired final volume. Each well was loaded with 10 μM freshly prepared Aβ_42_, either alone or mixed with sideridiol, and 25 μM ThT (Sigma-Aldrich, St. Louis, MO, USA) and the plates were sealed with microplate covers. Fluorescence measurements were performed through the bottom using a 444 nm filter for excitation and a 484 nm filter for emission. ThT diluted in HEPES was used for background measurement. Each experiment lasted 40 h with ThT fluorescence collected every 15 min after 10 s of agitation at 270 rpm; at least three independent experiments per sample were conducted. Background fluorescence was subtracted from the sample fluorescence at each time point, and the data normalization was performed on a scale of 0 to 100 arbitrary units. Standard deviation (SD) for each point was calculated, and error bars in ThT fluorescence emission spectra represent the SD of the triplicates. The R statistical language [45], along with packages ggplot2, dplyr, ggthemes, extrafont, and ggpmisc, integrated into the RStudio development environment were utilized for data visualization [46].

### 2.13. Molecular Dynamics Simulation

To examine the interaction between Aβ_42_ and sideridiol and the binding affinity of the complex, the publicly available software AutoDock Vina was used for Protein-Ligand Docking [47]. The NMR-derived structure of the oligomer of the Aβ peptide and the 3D structure of the ligand sideridiol were retrieved from the Protein Data Bank (PDB ID:2BEG) [48,49] and PubChem (PubChem CID: 12315541) [50], respectively. The conformation of the complex with the lowest binding affinity was used for the following computational experiment as it is considered the most energetically favorable.

Molecular dynamics simulation was conducted with the GROMACS software package (University of Groningen, Groningen, The Netherlands), version 2018.1 [51,52], to further examine the interaction between Aβ_42_ and sideridiol. The topologies for the oligomeric state of the Aβ_42_ peptide and the ligand were generated with the all-atom additive CHARMM36 protein force field [53] and the CHARMM General Force Field (CGenFF) [54], respectively. The solvated, neutralized system was subsequently energy-minimized with the steepest descent algorithm. Two phases of equilibration were performed; the first one was carried out under a constant volume (NVT) ensemble for 100 ps and temperature was maintained at 310 K, with the employment of V-rescale thermostat [55]—a modified Berendsen thermostat, suitable for Protein Non-Protein systems. The second phase was performed under a constant pressure (NPT) ensemble for 100 ps, and pressure was isotropically maintained at 1.013 bar (1 atm) with the use of Berendsen barostat [56]. Simulations were conducted for 500 ns at 310 K without position restraints using the Parrinello–Rahman barostat [57,58] and leap-frog algorithm with a 2-fs time step.

Tools provided by the GROMACS software package were used to perform the trajectory conversion and the analysis of the data produced by the simulation. Our analysis focused on the investigation of the effect of sideridiol on the stability of Aβ_42_ oligomer. For that purpose, the number of residues assigned to secondary structural elements, such as beta-sheets and helices, was calculated. The Root Mean Square Deviation (RMSD) and the Root Mean Square Fluctuation (RMSF) metrics were employed to evaluate the structural stability of the oligomerized Aβ_42_ peptide.

### 2.14. Statistical Analysis

All statistical analyses were performed with GraphPad Prism Software 8 (GraphPad Software, Inc., La Jolla, CA, USA) and Microsoft Office 365 Excel (Microsoft Corporation, Redmond, WA, USA) software packages. For analyses comparing the mean of two groups, we used the parametric two-tailed Student’s *t*-test, provided that each group comprised at least 10 values, with the exception of the dot blot densitometries, where we analyzed values from 3 independent experiments (2 independent experiments for SCP (EtOAc) sample). For paralysis curves, we used the log-rank Mantel–Cox test that compares whether two curves differ significantly. *n* in the paralysis figures represents the number of paralyzed animals. All bar charts indicate the mean of the independent experiments ± standard error of the means (SEM), with the exception of the ThT kinetic assays, where error bars in ThT fluorescence emission spectra represent the SD of the values obtained from the respective independent experiments.

## 3. Results

### 3.1. Phytochemical Composition of the Different SCP Extracts

We performed extractions from the aerial parts of SCP using EtOAc and then MeOH (Figure 1). This resulted in two extracts of completely distinct phytochemical composition; the mid-polar EtOAc extract was rich in diterpenes, and the polar MeOH extract was rich in phenylpropanoid esters/glycosides and glycosylated flavones. More specifically, the LC-MS analysis of the SCP (EtOAc) extract showed the presence of at least 11 compounds, of which we could identify only 5, 1 iridoid glycoside and 4 kaurane/kaurene diterpenes (Table 1, Figure S1). The iridoid ajugoside was in unquantifiable amounts, but we have recently reported its presence in SCP [15]. The compounds present in high concentrations were the kaurenes siderol (55.6%), sideridiol (22.8%), and 3,7,18-triacetyl-foliol (5.7%). The epoxykaurane sideroxol was present only in 1.2%. Their presence in SCP is demonstrated for the first time.

LC-MS analysis of the SCP (MeOH) extract demonstrated the presence of 16 peaks, among which 2, peaks 10 and 16, were a mixture of compounds (Table 2, Figure S2). We identified and determined two iridoid glycosides, melittoside and 7-*O*-acetyl-8-epi-loganic acid, with a total percentage of 4.85%. The two most abundant compound categories were flavone glycosides (31.70%) and phenylpropanoid/phenylethanoid glycosides (30.59%). The former flavone category includes many allosyl–glucosyl–hypolaetin and isoscutellarein derivatives which may also be acetylated or methylated. Hypolaetin and isoscutellarein are penta- and tetra-hydroxy flavones. Verbascoside was the major ingredient at a percentage of 17.31% and the most representative of the phenylpropanoid/phenylethanoid glycosides that has been isolated and undoubtedly characterized [15]. The second ingredient in terms of abundance was the 5-*O*-caffeoylquinic acid ester, chlorogenic acid (11.48%).

### 3.2. SCP Extracts Reduce Aβ Toxicity and Aggregation

**Figure 2:**
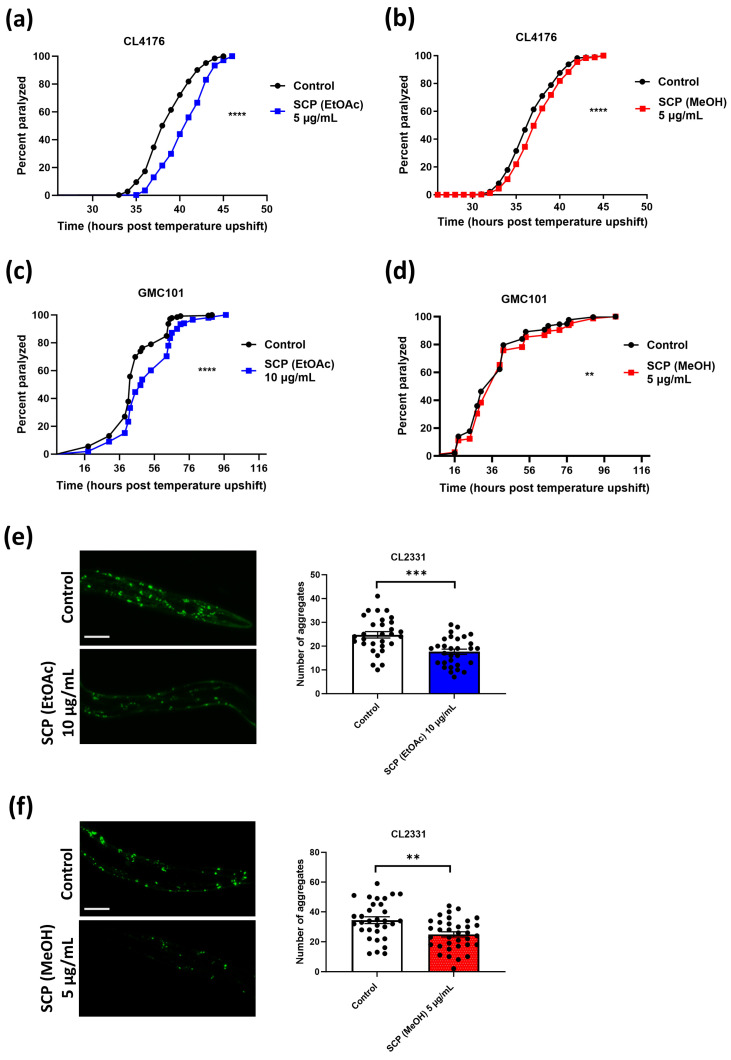
Treatment with SCP extracts confers protection against amyloid beta (Aβ) toxicity. Paralysis curves of (**a**,**b**) CL4176 and (**c**,**d**) GMC101 animals treated with SCP (**a**,**c**) (EtOAc) extract (5 μg/mL in (**a**) and 10 μg/mL in (**c**)); (**b**,**d**) (MeOH) extract (5 μg/mL) or control (DMSO). (**a**) Control: mean = 38.67 ± 0.67, *n* = 961; 5 μg/mL SCP (EtOAc) extract: mean = 40.67 ± 1.45, *n* = 901, three independent experiments, **** *p* < 0.001. (**b**) Control: mean = 36.86 ± 0.42, *n* = 4143; 5 μg/mL SCP (MeOH) extract: mean = 38.36 ± 0.54, *n* = 4426, 14 independent experiments, **** *p* < 0.0001. (**c**) Control: mean = 43.50 ± 0.87, *n* = 518; 10 μg/mL SCP (EtOAc) extract: mean = 53.50 ± 5.81, *n* = 500, four independent experiments, **** *p* < 0.0001. (**d**) Control: mean = 38 ± 3.37, *n* = 607; 5 μg/mL SCP (MeOH) extract: mean = 41 ± 5.32, *n* = 687, four independent experiments, ** *p* < 0.01. Median paralysis values are expressed as mean ± SEM. *n* denotes the number of animals that were paralyzed. Curves are the pooled result of the respective independent experiments. For paralysis experiments, the log-rank Mantel–Cox test was used. (**e**,**f**) Representative fluorescence micrographs of CL2331 animals expressing the human Aβ_3–42_ peptide conjugated to GFP treated with (**e**) 10 μg/mL SCP (EtOAc) extract (*n* = 30) or control (*n* = 30) and (**f**) 5 μg/mL SCP (MeOH) extract (*n* = 35) or control (*n* = 32) from egg hatching throughout the experiment and the relative quantification of amyloid deposits in the anterior area of the animals (three independent experiments). N represents the number of animals quantified. Each dot on the figure represents a quantified animal. The values are reported as the mean number of aggregates (in all quantified animals) ±SEM. ** *p* < 0.01, *** *p* < 0.001 (two-tailed Student’s *t*-test). Scale bar: 5 μm.

We then sought to investigate the potential effects of SCP extracts against Aβ toxicity by taking advantage of established *C. elegans* strains that are widely used as models for AD [36]. The two different extracts were initially tested in the transgenic nematode strain CL4176 that expresses the human Aβ_1–42_ peptide in its body wall muscle cells in a temperature-dependent manner. Upon temperature upshift, Aβ is expressed, resulting in Aβ aggregation and subsequent paralysis of the animals [60]. Various concentrations were applied (5, 10, and 20 μg/mL for SCP (EtOAc); Figure 2a and Figure S3a and 1 and 5 μg/mL for SCP (MeOH); Figure 2b and Figure S3b). Both extracts conferred delay of the Aβ-induced paralysis in treated animals; 5 μg/mL was the lowest concentration where we detected positive results for both extracts (Figure 2a,b).

To further validate the effects of both extracts in the reduction in Aβ toxicity, we tested both extracts in a second transgenic nematode strain, namely, GMC101, where the human Aβ_1–42_ peptide is constitutively expressed under a muscle-specific promoter, resulting in age-progressive paralysis [61]. Similarly to the CL4176 results, GMC101 animals were paralyzed significantly more slowly in the presence of both extracts (Figure 2c,d; 10 μg/mL SCP (EtOAc) extract was used in these experiments since this concentration gave more pronounced results in the CL4176 strain).

To visualize the Aβ aggregates in vivo, we took advantage of the CL2331 transgenic nematode strain. This strain expresses the human Aβ_3–42_ peptide conjugated with green fluorescent protein (GFP) in its body wall muscle cells and gradually accumulates Aβ aggregates that can be detected through confocal microscopy [62]. We scored reduced Aβ deposits in the presence of both SCP (EtOAc) and (MeOH) extracts (Figure 2e,f). In total, our results reveal a protective effect of SCP extracts (both EtOAc and MeOH) against Aβ aggregation and toxicity.

### 3.3. Sideridiol Contributes against Aβ Toxicity and Aggregation

We then sought to investigate which of the compounds contained in SCP (EtOAc) extract is responsible for its positive action against Aβ toxicity. As the SCP (EtOAc) extract gave more pronounced results in all tested concentrations, we applied a fractionation process with multiple chromatographic steps. We successfully isolated and characterized the two most abundant diterpenes, namely, siderol and sideridiol, in sufficient amounts (for isolation procedures and structures; Figure 1). Their presence in the endemic SCP is reported here for the first time, although it is not an uncommon finding in the genus [11].

**Figure 3:**
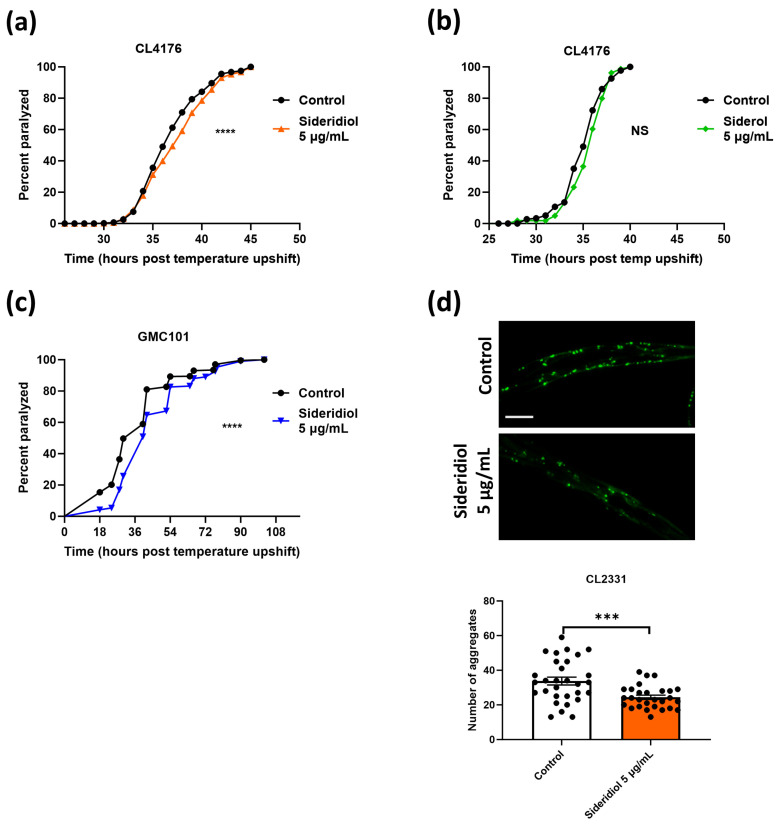
Sideridiol is the main contributor of SCP (EtOAc) extract effects against Aβ toxicity. Paralysis curves of (**a**,**b**) CL4176 and (**c**) GMC101 animals treated with (**a**,**c**) 5 μg/mL sideridiol and (**b**) 5 μg/mL siderol or control (DMSO). (**a**) Control: mean = 37.8 ± 0.86, *n* = 1478; 5 μg/mL sideridiol: mean = 39.20 ± 0.97, *n* = 1208, five independent experiments, **** *p* < 0.0001. (**b**) Control: mean = 36, *n* = 177; 5 μg/mL siderol: mean = 36, *n* = 159, 1 experiment, NS (not significant). (**c**) Control: mean = 37.33 ± 4.67, *n* = 475; 5 μg/mL sideridiol: mean = 49.33 ± 4.67, *n* = 501, three independent experiments, **** *p* < 0.0001. Median paralysis values are expressed as mean ± SEM. *n* denotes the number of animals that were paralyzed. Curves are the pooled result of the respective independent experiments. For paralysis experiments, the log-rank Mantel–Cox test was used. (**d**) Representative fluorescence micrographs of CL2331 animals expressing the human Aβ_3–42_ peptide conjugated to GFP treated with 5 μg/mL sideridiol (*n* = 28) or control (*n* = 30) from egg hatching throughout the experiment and the relative quantification of amyloid deposits in the anterior area of the animals (three independent experiments). *n* represents the number of animals quantified. Each dot on the figure represents a quantified animal. The values are reported as the mean number of aggregates (in all quantified animals) ±SEM. *** *p* < 0.001 (two-tailed Student’s *t*-test). Scale bar: 5 μm.

Various concentrations of both compounds were tested in CL4176 strain (Figure 3a,b and Figure S4a,b). Paralysis assays in CL4176 animals revealed a significantly slower paralysis rate in the presence of 5 μg/mL sideridiol (Figure 3a), but notably not in the presence of siderol (Figure 3b). Sideridiol also delayed paralysis at the 10 μg/mL concentration (Figure S4a), whereas siderol did not give positive results at any tested concentration (Figure S4b). Siderol and sideridiol are both ent-kaurenes, with the only difference between them being that siderol is an acetyl ester of sideridiol at position 7; obviously, the diol is necessary for the biological activity.

We further tested the effects of sideridiol in GMC101 animals and showed a similar positive effect (Figure 3c). In vivo visualization of the Aβ aggregates in the CL2331 strain revealed that treatment with 5 μg/mL of sideridiol reduced the Aβ aggregate formation (Figure 3d). In total, our results reveal that the protective effects of SCP (EtOAc) extract are at least partially due to sideridiol.

### 3.4. Verbascoside Contributes against Aβ Toxicity and Aggregation

**Figure 4:**
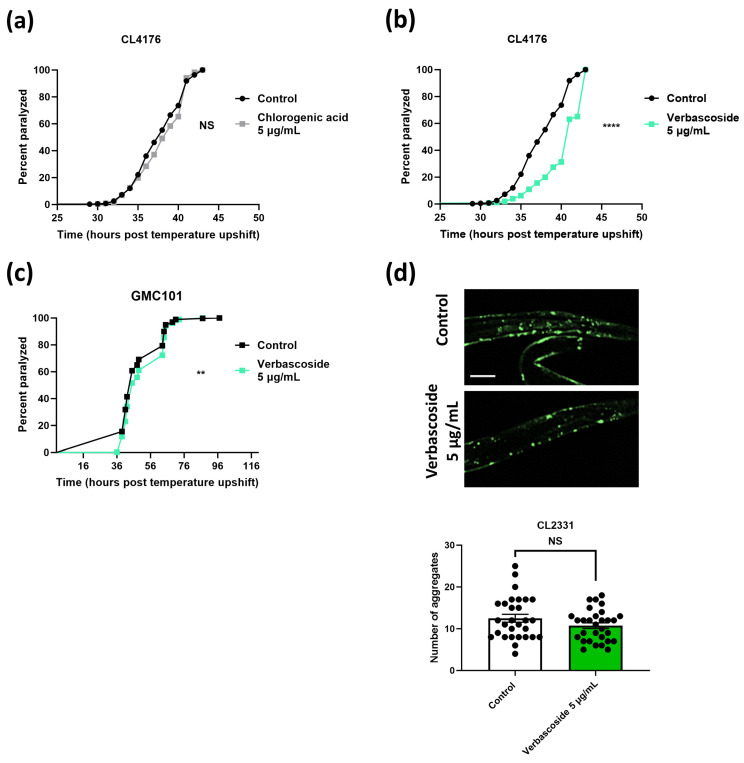
Verbascoside is the main contributor of SCP (MeOH) extract effects against Aβ toxicity. Paralysis curves of (**a**,**b**) CL4176 and (**c**) GMC101 animals treated with (**a**) 5 μg/mL cholorogenic acid and (**b**,**c**) 5 μg/mL verbascoside or control (H_2_O). (**a**) Control: mean = 38 ± 1, *n* = 383; 5 μg/mL cholorogenic acid: mean = 39.5 ± 1.5, *n* = 358, two independent experiments, NS. (**b**) Control: 38 ± 1, *n* = 383; 5 μg/mL verbascoside: mean = 42 ± 1, *n* = 255, two independent experiments, **** *p* < 0.0001. (**c**) Control: mean = 45 ± 0, *n* = 398; 5 μg/mL verbascoside: mean = 51.33 ± 6.33, *n* = 440, three independent experiments, ** *p* < 0.01. Median paralysis values are expressed as mean ± SEM. *n* denotes the number of animals that were paralyzed. Curves are the pooled result of the respective independent experiments. For paralysis experiments, the log-rank Mantel–Cox test was used. (**d**) Representative fluorescence micrographs of CL2331 animals expressing the human Aβ_3–42_ peptide conjugated to GFP treated with 5 μg/mL verbascoside (*n* = 30) or control (*n* = 29) from egg hatching throughout the experiment and the relative quantification of amyloid deposits in the anterior area of the animals (three independent experiments). *n* represents the number of animals quantified. Each dot on the figure represents a quantified animal. The values are reported as the mean number of aggregates (in all quantified animals) ±SEM. NS (two-tailed Student’s *t*-test). Scale bar: 5 μm.

Our results also revealed beneficial effects upon SCP (MeOH) extract supplementation (Figure 2b,d,f), albeit to a lesser extent compared to the SCP (EtOAc) extract. We subfractionated the SCP (MeOH) extract and ended up with the two most abundant compounds, namely, verbascoside and chlorogenic acid (for isolation procedures and structures; Figure 1). Paralysis assays in CL4176 animals revealed decelerated paralysis rates in animals treated with verbascoside but not chlorogenic acid (at 5 μg/mL concentration) (Figure 4a,b). Higher concentrations of chlorogenic acid (10 and 20 μg/mL) were also tested, and a reduced paralysis rate was observed at the highest concentration (Figure S5a). Nevertheless, although it was statistically significant, it was minor (less than an hour of deceleration compared to the control animals) compared to the one induced by verbascoside (~3 h at 5 and 10 μg/mL concentrations) (Figure S5b). We therefore further investigated the effects of verbascoside.

Treatment of GMC101 animals with verbascoside resulted in a reduced paralysis rate (Figure 4c), although the effect was not as pronounced as with sideridiol (5 μg/mL; Figure 3c). Nevertheless, in vivo visualization of the Aβ aggregates in the CL2331 strain did not reveal a reduction in Aβ aggregate formation, although a tendency was shown (Figure 4d). In total, our results suggest that the protective effects of SCP (MeOH) extract are at least partially due to verbascoside.

### 3.5. Combination of Sideridiol and Verbascoside Do Not Act Synergistically

**Figure 5:**
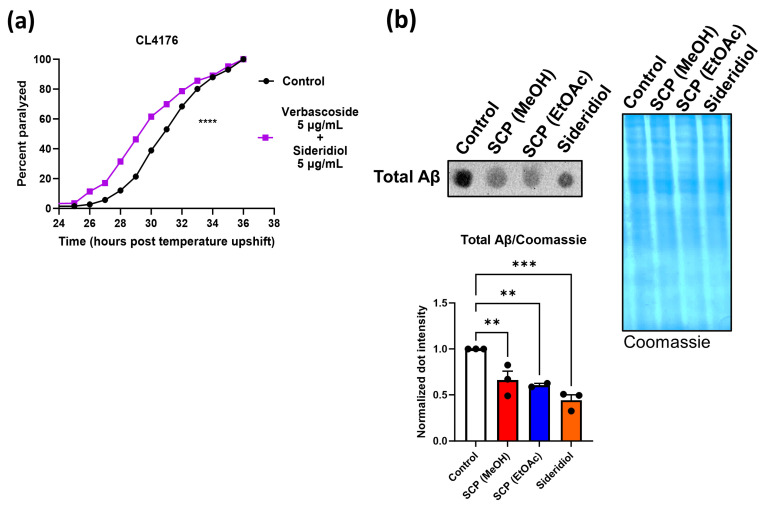
Treatment with SCP extracts or sideridiol results in reduced total Aβ protein levels. (**a**) Paralysis curves of CL4176 animals treated with 5 μg/mL verbascoside and 5 μg/mL sideridiol or control (DMSO). Control: mean = 31, *n* = 332; 5 μg/mL verbascoside/sideridiol: mean = 30, *n* = 229, one independent experiment, **** *p* < 0.0001. *n* denotes the number of animals that were paralyzed. For paralysis experiments, the log-rank Mantel–Cox test was used. (**b**) Dot blot analysis (with representative blots from three independent experiments with the exception of SCP (EtOAc), where two independent experiments were performed) of total Aβ levels in CL4176 animals treated with 5 μg/mL EtOAc extract, 5 μg/mL MeOH extract, 5 μg/mL sideridiol, or control (DMSO), collected when 50% of the control population was paralyzed. A gel stained with Coomassie brilliant blue was used as a loading control in each experiment. The mean value of signal of the protein of interest to the Coomassie signal in control animals was set to 1. Quantification that appears besides the blots represents the mean of three independent experiments ± SEM. ** *p* < 0.01, *** *p* < 0.001 (two-tailed Student’s *t*-test).

Given that both sideridiol and verbascoside gave positive results in paralysis assays, we hypothesized that simultaneous treatment with both compounds might result in additive positive effects. Nevertheless, the combination turned out to be deleterious at least within the range of the used concentrations (Figure 5a). Finally, since both SCP extracts (EtOAc and MeOH) and sideridiol were the only treatments that decelerated the paralysis rate of both AD strains (CL4176 and GMC101) and reduced Aβ-GFP aggregates in the CL2331 strain, we also examined the total Aβ protein levels in CL4176 animals under these treatments. SCP (EtOAc) extract, SCP (MeOH) extract, and sideridiol treatment resulted in significantly reduced total Aβ levels (Figure 5b).

### 3.6. Sideridiol Affects the Amyloidogenicity of Aβ_42_

**Figure 6:**
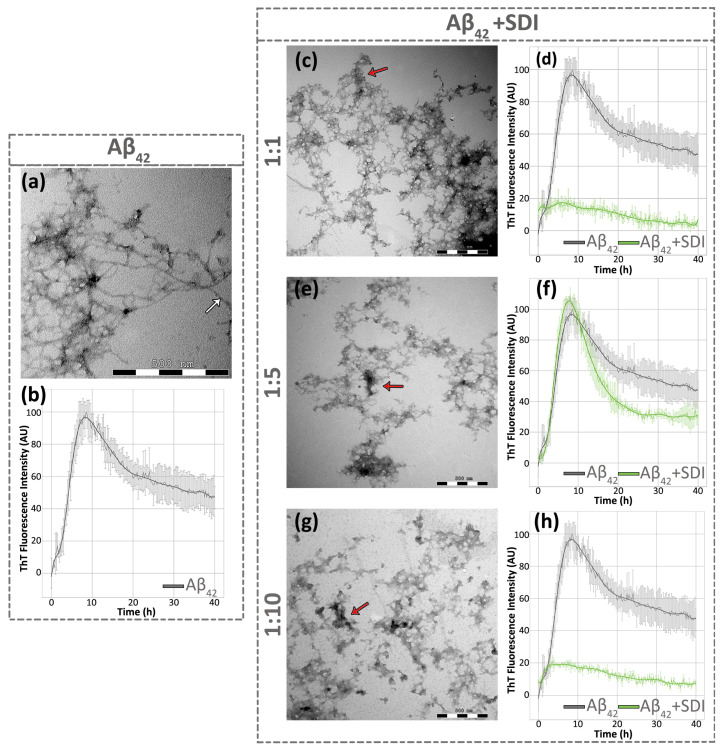
Sideridiol delays Aβ_42_ aggregation. (**a**,**c**,**e**,**g**) Transmission electron micrographs of (**a**) Aβ_42_ peptide alone and (**c**,**e**,**g**) Aβ_42_ peptide co-incubated with sideridiol (Aβ_42_ + SDI) in (**c**) 1:1, (**e**) 1:5, and (**g**) 1:10 ratios. The effect of sideridiol on Aβ_42_ fibrilization is evident, as the network of (**a**) Aβ_42_ amyloid-like fibrils is no longer observed in the presence of (**c**,**e**,**g**) sideridiol. White arrows point to single fibrils and red arrows point to amorphous aggregates. Scale bar: 500 nm. (**b**,**d**,**f**,**h**) ThT fluorescence emission spectrum of (**b**) Aβ_42_ peptide alone as a control and (**d**,**f**,**h**) Aβ_42_ peptide co-incubated with sideridiol for 40 h in (**d**) 1:1; (**f**) 1:5; and (**h**) 1:10 ratios. ThT fluorescence analysis supports the inhibitory effect of sideridiol on Aβ_42_ fibrillization, with notable reduction in fluorescence intensity at different ratios. Aβ_42_ peptide and the co-incubation with sideridiol are represented by a grey and a green line, respectively.

Among the tested pure compounds, sideridiol exhibited consistently positive outcomes across all assays, including those assessing Aβ_42_ aggregates. To further investigate the potential of sideridiol in inhibiting Aβ_42_ aggregation, additional in vitro studies were conducted. To directly visualize the changes in the morphology of Aβ_42_ aggregates upon treatment with sideridiol, we utilized TEM after negative staining. More specifically, sideridiol significantly affected the aggregation process of Aβ_42_ after 7 days of incubation. When Aβ_42_ peptide was individually incubated, it self-assembled into mature, straight, and unbranched fibrils with indefinite length and a diameter of approximately 80–90 Å, a typical morphology of amyloid fibrils [63] (Figure 6a). However, TEM micrographs following co-incubation of Aβ_42_ and sideridiol in three different ratios (Aβ_42_:Sideridiol: 1:1, 1:5, 1:10) revealed the absence of elongated and unbranched fibrils (Figure 6c,e,g). Instead, amorphous and prefibrillar aggregates of indeterminate diameter were observed.

To complement our TEM observations, we utilized ThT kinetic assay, a widely-used technique for its ability to detect amyloid fibrils [64]. ThT specifically binds to amyloid fibrils, resulting in a strong fluorescence signal. This approach allowed us to monitor the kinetics of Aβ_42_ fibrilization in the presence of different conditions of co-incubation with sideridiol. ThT fluorescence measurements at various time points confirmed the formation of amyloid-like fibrils by Aβ_42_ at approximately eight hours (Figure 6b). However, co-incubation of Aβ_42_ with sideridiol led to a significant decrease in ThT emission (Figure 6d,f,h). More specifically, ThT kinetics demonstrated that sideridiol induced a 78.75% decrease in the ThT fluorescence intensity at the 1:10 ratio (Figure 6h), 34.65% at the 1:5 ratio (Figure 6f), and 80.47% at the 1:1 ratio (Figure 6d). Notably, for the 1:1 and 1:10 ratios, the reduction was evident within the first 5 h, while in the 1:5 ratio, a significant reduction was observed only after 20 h. Hence, ThT fluorescence analysis provided data supporting the inhibitory effect of sideridiol on Aβ_42_ aggregation. Overall, the combination of TEM and ThT kinetic assays allowed for a comprehensive investigation of the impact of sideridiol on Aβ_42_ fibrillization, providing insights into the modulation of amyloid fibril formation by this compound.

### 3.7. Sideridiol Affects the Tertiary Structural Stability of the Aβ_42_ Oligomer

**Figure 7:**
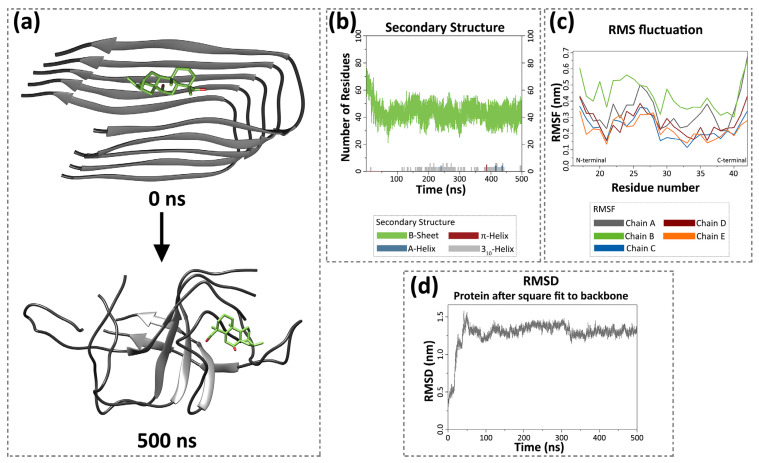
Results of molecular dynamics simulation of Aβ_42_ oligomer with sideridiol. (**a**) Simulation frames of the Aβ_42_ oligomer in the presence of sideridiol at 0 ns and 500 ns. The Aβ_42_ peptide adopts a different conformation at the end of the simulation (500 ns). Aβ_42_ peptide is coloured grey, while sideridiol is represented in light green. (**b**) The number of residues assigned to B-sheet (green), A-helix (blue), π-helix (red), and 3_10_-helix (grey) during the simulation, according to the DSSP calculations. (**c**) The Root Mean Square Fluctuation (RMSF) per residue for the five chains of the Aβ_42_ oligomer. (**d**) The Root Mean Square Deviation (RMSD) of Aβ_42_ oligomer throughout the simulation.

To gain insights into the interaction between the oligomeric structure of Aβ_42_ and sideridiol, a molecular dynamics simulation of 500 ns was conducted (Figure 7). By examining the snapshots acquired for the complex of the Aβ_42_ peptide with sideridiol from the beginning (0 ns) until the end of the simulation (500 ns), the small molecule manages to maintain its topological affinity with the peptide since it can be observed that the interaction of sideridiol with the peptide was constant throughout the simulation. In addition, the Aβ_42_ peptide eventually acquires a different structural conformation than the one that characterizes its pathological state (Figure 7a, 0 ns), whereby the chains appear partially disordered (Figure 7a, 500 ns).

Beta-strand is the secondary structure that has been intimately associated with the pathological nature of amyloids and especially with the transition of soluble helical monomers to insoluble, toxic oligomers [65]. The DSSP (Dictionary of Protein and Secondary Structure) algorithm is among the most common and established methods that can determine the secondary structure and geometric characteristics of proteins [66,67]. Thus, DSSP was implemented in the GROMACS suite to draw the secondary structure plot as a function of the simulation time. This graph offers insight into the conformational changes of the Aβ_42_ peptide as it defines the number of residues assigned to beta-strands or helices (α-, π-, or 3_10_-helices), as well as the retention of each secondary structure during the simulation. The formation of helices is of great importance as this specific structural element is usually observed in the non-pathological, soluble form of the Aβ_42_ peptide [68]. As shown in Figure 7b, the interaction of the oligomerized Aβ_42_ with sideridiol resulted in a steep decrease in the number of amino acid residues engaged in beta-strands within the Aβ_42_ peptide (green line). The number of the residues in beta-strands was reduced by 31% when comparing the average numbers between the first and the last 5 ns of the simulation, and the compound transiently facilitated the formation of helical structures. More thoroughly, amino acid residues remained assigned to the 3_10_-helix structure (grey line, Figure 7b) for longer periods during the simulation, while α- and π-helices were only formed for a few nanoseconds (blue and red lines, respectively, Figure 7b).

The analysis of RMS fluctuation has been utilized to evaluate the flexibility of the peptide structure on a per-amino-acid-residue basis in every chain of the pentamer. Specifically, it can be assessed as to whether some or all five chains of the peptides are more flexible, relative to their original state, as higher RMSF values indicate higher flexibility. Regarding the interaction between sideridiol and the oligomer of the Aβ_42_ peptide, the chain of the peptide that showed the greatest mobility, and therefore was the most flexible, was chain B (Figure 7c, green line). More specifically, chain B was affected along its entire length by the interaction of the Aβ_42_ peptide with the ligand sideridiol. The RMSF value for the C-terminus of chain B was higher than the value of the N-terminus, resulting in the conclusion that the C-terminus was more flexible and the one being mostly displaced. It can also be noted that chain A exhibited changes in its structural integrity, and subsequently, it was flexible as well. The C-terminus of both chains A and B was characterized by fluctuations greater than 0.6 nm. On the other hand, chains C, D, and E showed lower values of RMSF throughout their entire length compared to the other two chains. Hence, chains C, D, and E of the oligomerized Aβ_42_ peptide did not change significantly due to their interaction with the ligand sideridiol.

A further assessment of the conformational alterations of the oligomer due to its interaction with sideridiol was conducted through RMSD calculations. Elevated RMSD values signify a less stable structure, affirming the potency of the compound to influence the stability of the Aβ_42_ oligomer. Sideridiol caused a sharp increase in the RMSD value in the first 50 ns up to 1.5 nm, and it was maintained above 1 nm throughout the simulation, showing that the overall structure of the oligomer was destabilized, thus demonstrating the effect of sideridiol on the initial conformation of the peptide (Figure 7d). Ultimately, our molecular dynamics simulation revealed that sideridiol affected the tertiary structural stability of the Aβ_42_ oligomer, offering valuable insights that align with and provide a rationale for the observed in vitro results.

### 3.8. Lack of Toxicity of SCP Extracts, Sideridiol and Verbascoside

Finally, we sought to evaluate the potential toxicity of the various extracts and pure compounds that resulted in protection against Aβ toxicity, namely, SCP (MeOH and EtOAc) extracts, sideridiol and verbascoside. We thus took advantage of the nematode *C. elegans* that has been suggested as a suitable model for toxicity testing [69,70]. Various phenotypic characteristics of the nematode have been shown to be affected upon toxic exposure [71]. We monitored the following phenotypic characteristics in wt nematodes: developmental timing, fertility, egg lethality, pharyngeal pumping, and defecation rate (Table 3, Table 4, Table 5 and Table 6). The animals were grown on each compound since hatching; no alterations in development advocate for lack of toxicity. The outcome of the phenotypic analysis showed that the SCP (EtOAc) extract is not harmful for the animal (Table 3) since, with the exception of the accelerated defecation rate and the enhanced pharyngeal pumping, the remaining phenotypic characteristics remained unaltered. It is also noteworthy that even the observed alterations are considered beneficial. More specifically, accelerated defecation rate is a trait associated with faster detoxification and thus better animal fitness [72]. Likewise, enhanced pharyngeal pumping has been observed in long-lived animals [71,73], whereas pharyngeal pumping usually diminishes upon exposure to toxicants [69].

Likewise, SCP (MeOH) extract only affected the defecation rate of the animals (Table 4).

More importantly, treatment with sideridiol did not induce any changes in the treated animals, consistent with the lack of toxicity (Table 5).

Finally, phenotypic analysis of animals treated with verbascoside revealed a significantly decreased defecation rate with no other changes, thus coinciding for a low but still existing toxicity potential (Table 6).

In total, our phenotypic analysis did not reveal any severe alterations of the various characteristics (with the exception of verbascoside in the specific concentration used), thus, coinciding for the lack of toxicity of the involved extracts and compounds.

## 4. Discussion

AD is a detrimental neurodegenerative disease lacking an effective cure. A lot of effort has been put into the identification of synthetic or natural compounds, including antioxidants (due to the strong link between oxidative stress and AD [18]) that can be used in preventive or therapeutic strategies against disease progression. With regard to bioactive natural products, a lot of attention has been paid to compounds found in edible species since they are believed to be capable of altering the fate of a disease at early stages when the disease is not yet detectable. More specifically, natural products with antioxidant and neuroprotective properties against AD represent a highly developing field with the potential to offer valuable drugs or nutraceuticals [5]. Although various groups have revealed the neuroprotective properties, mainly of *S. scardica* extracts in cellular and animal models [23,25,26,28,30] as well as in humans [33,34,35], very scarce data exist on another *Sideritis* taxon, SCP, and its neuroprotective properties. Moreover, it is noteworthy that, so far, only *Sideritis* spp. extracts have been tested, and the responsible distinct compounds have not been identified. In this paper, we report the characterization of SCP (EtOAc) and (MeOH) extracts (with LC-MS) and we are the first to reveal their protective effects against Aβ aggregation. More importantly, we have investigated the potential neuroprotective properties of their most abundant ingredients (two ent-15-kaurene diterpenes, namely, siderol and sideridiol, and two phenolic derivatives, i.e., chlorogenic acid and verbascoside) and we identified at least two pure compounds, namely, sideridiol and verbascoside, being responsible for the beneficial effects. Finally, through in vitro experiments, we revealed that sideridiol affects the amyloidogenicity of Aβ_42_ peptide, while in silico studies suggested that it also affects the tertiary structural stability of the Aβ_42_ oligomer.

By taking advantage of three different established *C. elegans* strains, we revealed that both SCP (EtOAc) and (MeOH) extracts can reduce Aβ toxicity and aggregation. Similar results have been shown for the mid-polar extracts of *S. scardica* since they conferred a reduction in the number of Aβ plaques in the CL2006 strain, as well as a significant delay of the paralysis of CL4176 animals [27]. In accordance, analogous results were also collected in a mouse model. More specifically, daily oral administration of *S. scardica* extract (and *S. euboea* extract) to aged, non-transgenic, and APP-transgenic mice resulted in enhanced cognition along with a significant reduction in the amyloid plaque burden in transgenic mice [28]. We have revealed a reduced paralysis rate in two different nematode strains, namely, CL4176, that becomes paralyzed in a few hours (acute paralysis [60]), and GMC101, that manifests age-progressive paralysis [61]. More importantly, we have verified a significantly reduced number of Aβ aggregates in the presence of both extracts in the CL2331 strain, where one can measure in vivo the aggregates of human Aβ peptide [62]. Notably, SCP extracts conferred protection in AD nematode models in significantly lower concentrations (ranging between 5 and 20 μg/mL) compared to similar work conducted with *S. scardica* extracts (ranging between 200 and 600 μg/mL [27]), thus suggesting a higher potential of SCP extracts against Aβ toxicity and aggregation. Since SCP possesses weaker antioxidant properties compared to *S. scardica* [15], the higher potential of the SCP extracts against Aβ toxicity and aggregation that we report here is probably due to additional beneficial properties. Indeed, our in vitro and in silico studies suggested anti-amyloidogenic properties of sideridiol, a major compound of SCP. Future studies regarding SCP extracts should focus on experimentation in higher eukaryotes to verify their neuroprotective potential.

Results derived from extracts are valuable and may lead to the production of potential herbal medicinal products and dietary supplements, but still, the identification of the specific bioactive compounds that are responsible for the observed outcomes is indispensable. Although a lot of work has been conducted on *S. scardica* extracts (water, mid-polar, etc. (references throughout the text)), the specific compounds responsible for the positive effects have never been identified so far. We therefore decided to identify the responsible bioactive compounds in the different SCP extracts, and we report that sideridiol is at least one of those compounds in SCP (EtOAc) extract and verbascoside in SCP (MeOH) extract. Although sideridiol has been previously tested for several activities, including lipid peroxidation inhibitory activity [74], inhibitory activity against acetyl cholinesterase (AChE) and butyrylcholinesterase (BChE) [74], antimicrobial activity [75], cytotoxic activity on cancer cell lines [37], and its potential to alter the feeding behavior of the final larval stage of the *Lepidoptera*, *Spodoptera frugiperda*, and *S. littoralis* [76], no significant effects were found [77]. In contrast, we report the anti-amyloidogenic potential of sideridiol for the first time. Our in vitro and in silico results suggest that sideridiol may be beneficial due to its ability to affect the amyloidogenicity of Aβ_42_ peptide and the tertiary structural stability of the Aβ_42_ oligomer and maybe not due to a potential antioxidant action (that has not previously been revealed anyway [74]). It is, however, noteworthy that since SCP (EtOAc) extract induced more pronounced results against Aβ-induced paralysis compared to the results given by sideridiol alone, this may imply that the SCP (EtOAc) extract may contain additional constituents with synergistic effects to sideridiol. Thus, we cannot rule out the potential synergistic action of other SCP (EtOAc) compounds and their possible link with antioxidation.

Antimicrobial [78,79] and anti-inflammatory [80] properties have been attributed to verbascoside (also known as acteoside). More importantly, verbascoside has been reported to protect human neuroblastoma SH-SY5Y cells against Aβ-induced cell injury [81], to confer neuroprotection against AD via the relief of endoplasmic reticulum stress in Aβ-exposed cells and APP/PS1 mice [82], and to exert neuroprotective effects in a model of Parkinson’s disease through induction of autophagy [83]. More recently, it was shown to alleviate learning and memory impairment in APP/PS1 transgenic mice through enhanced Aβ degradation and inhibition of tau hyperphosphorylation [84]. Although verbascoside attenuated the paralysis of both nematode models used in this study, which is in accordance with the above-mentioned neuroprotective properties, we did not detect significantly lower levels of Aβ aggregates in the CL2331 strain, although we report such a tendency. This discrepancy could be due to the different animal model used in our study, to another mechanism of action that does not necessarily go through the reduction in Aβ aggregation or the enhanced Aβ clearance, or to the lack of more extensive concentration-wise testing.

The neuroprotective properties of *S. scardica* extracts have been linked to various mechanisms of action. More specifically, various extracts have been shown to act as inhibitors of the uptake of serotonin, dopamine, and noradrenaline, which are neurotransmitters involved in various neurological disorders [23]. Ex vivo studies in rats have related the beneficial effects of *S. scardica* extract to the modulation of the AMPA receptor-dependent neurotransmission [24]. Moreover, levels of total and hyperphosphorylated tau have been shown to be reduced in AD neuronal cell culture models (PC12-htau and SH-SY5Y-AβPP cell lines) upon treatment with *S. scardica* extracts, while the non-amyloidogenic pathway of APP processing was shown to be induced [25]. Our results revealed that both SCP extracts and sideridiol affected the total levels of Aβ aggregates since they were found reduced in our in vivo model (CL2331 strain). These results were further verified via dot blot experiments. Our TEM results demonstrated the efficacy of sideridiol to halt the self-assembly of Aβ_42_ into amyloid-like fibrils. This observation was further verified by a significant reduction in ThT fluorescence intensity. Notably, these results align with the molecular dynamics simulation analysis, where an apparent conformational change in the secondary structure of the oligomeric form of Aβ_42_ was observed. It is possible that the interaction between the Aβ oligomeric state and sideridiol contributes to the reduction in the beta-strands connecting the monomers, ultimately leading to their removal. This, in turn, may result in less aggregated structures with reduced toxicity. These results pinpoint a potential mechanism of action for sideridiol against Aβ aggregation.

Natural bioactive compounds are considered to possess lower potential for negative side effects, while it is believed that they can be used to prevent, decelerate, or even treat various diseases, including AD [3,4]. The potential toxicity of the various extracts and pure compounds was evaluated by taking advantage of the nematode *C. elegans*, which has been suggested as a suitable model for toxicity testing [69]. Among the phenotypic characteristics tested, (a) defecation rate was accelerated by SCP (EtOAc) and (MeOH) extracts and decelerated by verbascoside; and (b) pharyngeal pumping rate was increased in the presence of SCP (EtOAc) extract. The lack of major negative alterations, along with the fact that the animals were grown on the tested compounds from hatching without presenting any developmental abnormalities, coincides with low toxicity. The accelerated defecation rate is associated with enhanced fitness of the animals, which permits faster detoxification [72]. Additionally, enhanced pharyngeal pumping has been linked to longevity [71,73], and various compounds positively affecting the lifespan of the animals have been shown to accelerate pharyngeal pumping rate [42,43,85]. Therefore, those alterations can be considered as beneficial ones. Verbascoside was the sole compound to produce a negative alteration of the defecation rate, which may reveal a small but still significant toxicity, at least in the used concentration.

With regard to human trials, only *S. scardica* extract has been tested, providing promising results for the improvement of cognitive performance either after an acute dose (probably due to a significantly altered response of the cerebral blood flow) or after a longer consumption (more likely due to a reduction in anxiety) [33]. Likewise, reduction in (mental) stress and improvement of stress tolerance was recorded in young volunteers (mean age: 38.70) after intake of a dietary supplement containing a herbal extract of *S. scardica* extract, vitamin B1, vitamin B6, folic acid, and vitamin B12 for 6 weeks [34]. Finally, volunteers suffering from MCI (a stage preceding AD manifestation) exhibited an improvement in mental performance when a combination of *S. scardica* and *B. monnieri* extracts was administered for 4 weeks [35]. Given that our results suggested SCP extracts to be more potent than *S. scardica* extracts (at least in AD nematode models), it would be worthwhile to set up human trials with SCP extracts.

Although our results are promising and give insights into the potential use of SCP (and its distinct compounds) against Aβ toxicity and aggregation, while also revealing the responsible bioactive natural products, our study also has a few limitations. First, our main experimental model was the nematode; therefore, more experimentation in neuronal cells and higher eukaryotes is needed. Low toxicity of the extracts and their active compounds was scored in *C. elegans*, but experiments in higher eukaryotes are needed. Although we further examined the most abundant constituents of SCP (EtOAc and MeOH) extracts, more compounds may also be responsible for the observed beneficial effects. Finally, there is an absolute need to design and conduct human trials to verify whether our promising results can be duplicated in humans, thus leading to reduced Aβ proteotoxicity and potentially to improvements in cognitive performance. Given that positive outcomes have already been reported for other *Sideritis* spp., and since our results suggested an even higher potential for the SCP extracts, we are optimistic regarding the effects of SCP extracts in humans.

## 5. Conclusions

In total, although a lot of work has been performed with various *Sideritis* spp. other than SCP in various cellular and animal models, as well as in human trials, our results reveal the potential of SCP against Aβ toxicity and aggregation. The identification of sideridiol and its anti-amyloidogenic properties suggest that every *Sideritis* spp. has its own arsenal of components worthy of investigation. Our results strengthen the notion that various *Sideritis* spp., including SCP, can be exploited as an extra source of bioactive extracts or compounds to develop functional foods, supplements, or even lead compounds for drug development against AD.

## Figures and Tables

**Table 1:** List of metabolites and their concentrations (mg sideridiol equivalents/g of dry plant extract, average values, *n* = 2) in the SCP (EtOAc) extract with reference to the identification method or previous references of the occurrence of the compound in the genus.

	t_R_ (min)	Ions (*m*/*z*) in Positive Ionization	M.W.	Compound	mg Sideridiol equiv./g Dry Extract
1	1.73	413 [M + Na]^+^	390	Ajugoside ^#^	nq
2	9.10	321 [M + H]^+^/343 [M + Na]^+^/ 663 [2M + Na]^+^	320	Sideroxol ^#^	6.08
3	13.41	385 [M + Na]^+^/747 [2M + Na]^+^	362	Unknown	14.12
4	14.63	327 [M + Na]^+^/631 [2M + Na]^+^	304	Unknown	nq
5	16.41	327 [M + Na]^+^/631 [2M + Na]^+^	304	Unknown	8.36
6	16.76	327 [M + Na]^+^/631 [2M + Na]^+^	304	Unknown	2.09
7	17.62	327 [M + Na]^+^, 631 [2M + Na]^+^	304	Sideridiol ^#^	111.59
8	25.53	427 [M + Na]^+^/809 [2M + H]^+^/831 [2M + Na]^+^	404	Unknown	47.44
9	33.50	369 [M + Na]^+^/715 [2M + Na]^+^	346	Siderol ^#^	272.89
10	40.82	469 [M + Na]^+^/915 [2M + Na]^+^	446	3,7,18-triacetyl-foliol ^1^	27.92
11	56.02	411 [M + Na]^+^/799 [2M + Na]^+^	388	Unknown	nq

^#^ Identification with isolated compounds, ^1^ Identification according to [59]. nq: not quantified.

**Table 2:** List of polar metabolites and their concentrations (mg rutin equivalents/g of dry plant extract, average values, *n* = 2) in the SCP (MeOH) extract.

	t_R_ (min)	Ions (*m*/*z*) in Negative Ionization	M.W.	Compound	mg Rutin equiv./g Dry Extract
1	3.24	191/383/533	-	Unknown	4.26
2	6.38	523 [M − H]^−^/583 [M + Hac − H]^−^/1047 [2M − H]^−^	524	Melittoside ^#^	4.37
3	17.92	191 (quinic acid)/353 [M − H]^−^/707 [2M − H]^−^/729 [2M − 2H + Na]^−^	354	Chlorogenic acid ^#^	10.35
4	23.91	417 [M − H]^−^	418	7-*O*-acetyl-8-epi-loganic acid ^#^	nq
5	26.79	392 [M − 2H]^2−^/785 [M − H]^−^	786	Unknown	3.23
6	27.11	377 [M − 2H]^2−^/755 [M − H]^−^	756	Forsythoside B ή Lavandulofolioside	9.55
7	28.06	311 [M − 2H]^2−^/623 [M − H]^−^/1248 [2M − H]^−^	624	Verbascoside ^#^	15.61
8	29.01	625 [M − H]^−^	626	All-Glc-HYP	2.69
9	32.36	769 [M − H]^−^	770	Allysonoside	3.43
10	34.03	637 [M_1_ − H]^−^/651 [M_2_ − H]^−^/623 [M_3_ − H]^−^	638 (M_1_) and 652 (M_2_) and 624 (M_3_)	M_1_: Leucoseptoside isomer/M_2_: AcO-All-Glc-ISC or AcO-All-Glc-LUT/M_3_: isoverbascoside ^#^	9.10
11	35.71	639 [M − H]^−^/1279 [2M − H]^−^	640	All-Glc-HYP-Me	nq
12	36.18	667 [M − H]^−^	668	AcO-All-Glc-HYP	8.79
13	42.43	651 [M − H]^−^/1303 [2M − H]^−^	652	AcO-All-Glc-ISC or AcO-All-Glc-LUT	9.62
14	43.26	681 [M − H]^−^/1364 [2M − H]^−^	682	AcO-All-Glc-HYP-Me	6.54
15	47.72	709 [M − H]^−^	710	(AcO)_2_-All-Glc-HYP	2.64
16	49.25	693 [M_1_ − H]^−^/723 [M_2_ − H]^−^	694 (M_1_) and 724 (M_2_)	M_1_: (AcO)_2_-All-Glc-ISC/M_2_: (AcO)_2_-All-Glc-HYP-Me	nq

^#^ Identification with isolated compounds. For more details on the identification see [15]. Abbreviations: nq: not quantified; AcO: *O*-acetyl; All: allosyl; Glc: glucoside; HYP: hypolaetin; ISC: isoscutellarein; LUT: luteolin; Me: methyl, Hac: acetic acid.

**Table 3:** Phenotypic analysis of wt animals treated with SCP (EtOAc) extract.

	Developmental Time ^a^	Fertility ^b^	Egg Lethality ^c^	Pharyngeal Pumping ^d^	Defecation ^e^
DMSO	51.27 ± 0.12	269.8 ± 11.81	0	279.4 ± 3.06	47.45 ± 0.71
10 μg/mL SCP (EtOAc)	52 ± 0.08	257.6 ± 13.70	0	288.2 ± 2.76 *	41.13 ± 0.57 ***

All assays were performed at 20 °C unless noted otherwise. ^a^ Duration of postembryonic development (hours from egg hatching to L4 stage). ^b^ Number of offspring per worm. ^c^ Number of eggs that did not hatch. ^d^ Pumps in 1 min on day 1 of adulthood. ^e^ Duration of defecation cycle in seconds at day 1 of adulthood. Error bars denote standard error of the mean ± SEM, *p* denotes *p* value of student’s *t*-test, * *p* < 0.05, *** *p* < 0.001.

**Table 4:** Phenotypic analysis of wt animals treated with SCP (MeOH) extract.

	Developmental Time ^a^	Fertility ^b^	Egg Lethality ^c^	Pharyngeal Pumping ^d^	Defecation ^e^
DMSO	52.15 ± 0.07	265.2 ± 8.81	0	311.64 ± 2.72	45.67 ± 0.50
5 μg/mL SCP (MeOH)	51.7 ± 0.08	276.3 ± 7.222	0	312.8 ± 2.52	43.36 ± 0.49 ***

All assays were performed at 20 °C unless noted otherwise. ^a^ Duration of postembryonic development (hours from egg hatching to L4 stage). ^b^ Number of offspring per worm. ^c^ Number of eggs that did not hatch. ^d^ Pumps in 1 min on day 1 of adulthood. ^e^ Duration of defecation cycle in seconds at day 1 of adulthood. Error bars denote standard error of the mean ± SEM, *p* denotes *p* value of student’s *t*-test *** *p* < 0.001.

**Table 5:** Phenotypic analysis of wt animals treated with sideridiol.

	Developmental Time ^a^	Fertility ^b^	Egg Lethality ^c^	Pharyngeal Pumping ^d^	Defecation ^e^
Control	51.08 ± 0.14	279.4 ± 7.81	0	292.9 ± 1.87	43.94 ± 0.74
5 μg/mL Sideridiol	51.41 ± 0.16	277.0 ± 8.62	0	289.4 ± 2.41	44.30 ± 0.43

All assays were performed at 20 °C unless noted otherwise. ^a^ Duration of postembryonic development (hours from egg hatching to L4 stage). ^b^ Number of offspring per worm. ^c^ Number of eggs that did not hatch. ^d^ Pumps in 1 min on day 1 of adulthood. ^e^ Duration of defecation cycle in seconds at day 1 of adulthood. Error bars denote standard error of the mean ± SEM.

**Table 6:** Phenotypic analysis of wt animals treated with verbascoside.

	Developmental Time ^a^	Fertility ^b^	Egg Lethality ^c^	Pharyngeal Pumping ^d^	Defecation ^e^
Control	51.67 ± 0.47	305.80 ± 18.12	0.75 ± 0.59	280.7 ± 3.92	42.73 ± 0.62
5 μg/mL Verbascoside	52.67 ± 0.47	299.80 ± 13.67	1.12 ± 0.41	289.2 ± 4.04	47.40 ± 0.39 ****

All assays were performed at 20 °C unless noted otherwise. ^a^ Duration of postembryonic development (hours from egg hatching to L4 stage). ^b^ Number of offspring per worm. ^c^ Number of eggs that did not hatch. ^d^ Pumps in 1 min on day 1 of adulthood. ^e^ Duration of defecation cycle in seconds at day 1 of adulthood. Error bars denote standard error of the mean ± SEM, *p* denotes *p* value of student’s *t*-test **** *p* < 0.0001.

## Data Availability

Data available within the article or Supplementary Materials.

## Supplementary Materials

The following supporting information can be downloaded at https://www.mdpi.com/article/10.3390/antiox13030261/s1: Figure S1: Total Ion chromatograph after positive (upper) and negative (lower) ionization of the SCP (EtOAc) extract; Figure S2: Total Ion chromatograph after positive (upper) and negative (lower) ionization of the SCP (MeOH) extract; Figure S3: Treatment with SCP extracts confers protection against Aβ toxicity; Figure S4: Sideridiol and not siderol is the main contributor of SCP (EtOAc) extract effects against Aβ toxicity; Figure S5: Verbascoside and not cholorogenic acid is the main contributor of SCP (MeOH) extract effects against Aβ toxicity.

## Abbreviations

AChE: acetyl cholinesterase; AD: Alzheimer’s disease; APP: amyloid precursor protein; Aβ: amyloid-beta; Aβ_42_: amyloid-beta amino acids 1 to 42; BchE: butyrylcholinesterase; *C. elegans*: *Caenorhabditis elegans*; DSSP: Dictionary of Protein and Secondary Structure; EtOAc: ethyl acetate; GFP: green fluorescent protein; HFIP: 1,1,1,3,3,3-Hexafluoro-2-propanol; MeOH: methanol; MCI: mild cognitive impairment; NGM: nematode growth medium; NS: not significant; SCP: *Sideritis clandestina* subsp. *peloponnesiaca*; SCC: *Sideritis clandestina* subsp. *clandestina*; RMSD: Root Mean Square Deviation; RMSF: Root Mean Square Fluctuation; SD: standard deviation; SEM: standard error of the means; TEM: Transmission Electron Microscopy; ThT: thioflavin T; wt: wild type.
