# Supplementary material for: Beneficial Effects of Sideritis clandestina Extracts and Sideridiol against Amyloid β Toxicity
Source: Antioxidants (Basel). 2024 Feb 21;13(3):261. doi: 10.3390/antiox13030261 (PMC10967505; doi:10.3390/antiox13030261)
Supplement: Supplementary file 1 [file antioxidants-13-00261-s001.zip › antioxidants-2799609-supplementary.pdf]

Supplementary file

Beneficial effects of *Sideritis clandestina* extracts and sideridiol against amyloid β toxicity

Anna Gioran [1,†], Yiorgos Paikopoulos [1,†,‡], Eleni Panagiotidou [1,2], Aikaterini E. I. Rizou [3,§], Georgia I. Nasi [3], Virginia D. Dimaki [4], Konstantina D. Vraila [3], Dimitra S. Bezantakou [3,§], Panagiotis M. Spatharas [3,‖], Nikos C. Papandreou [3], Vassiliki Magafa [4], Fotini N. Lamari [4], Vassiliki A. Iconomidou [3] and Niki Chondrogianni [1,*]

[1] Institute of Chemical Biology, National Hellenic Research Foundation, 48 Vassileos Constantinou Avenue, 11635 Athens, Greece; agioran@eie.gr (A.G.); yiorgos.paikopoulos@isas.de (Y.P.); epanagiotidou@eie.gr (E.P.)

[2] Department of Biochemistry and Biotechnology, University of Thessaly, 41334 Larissa, Greece

[3] Section of Cell Biology and Biophysics, Department of Biology, School of Sciences, National and Kapodistrian University of Athens, Panepistimiopolis, 15701 Athens, Greece; rizoukat@biol.uoa.gr (A.E.I.R.); gnasi@biol.uoa.gr (G.I.N.); vrailakon@biol.uoa.gr (K.D.V.); dimbez@biol.uoa.gr (D.S.B.); panspatharas@biol.uoa.gr (P.M.S.); npapand@biol.uoa.gr (N.C.P.); veconom@biol.uoa.gr (V.A.I.)

[4] Laboratory of Pharmacognosy and Chemistry of Natural Products, Department of Pharmacy, University of Patras, 26504 Patras, Greece; virnadimaki@upatras.gr (V.D.D.); magafa@upatras.gr (V.M.); flam@upatras.gr (F.N.L.)

[*] Correspondence: nikichon@eie.gr; Tel.: +30-210-7273768

[†] These authors contributed equally to this work.

[‡] Current address: Leibniz Institute for Analytical Sciences, ISAS e.V., 44227 Dortmund, Germany.

[§] Current address: Institute for Bio-Innovation, Biomedical Sciences Research Center "Alexander Fleming", 16672 Vari, Greece.

[‖] Current address: European Molecular Biology Laboratory, Hamburg Unit, Notkestrasse 85, 22607 Hamburg, Germany.

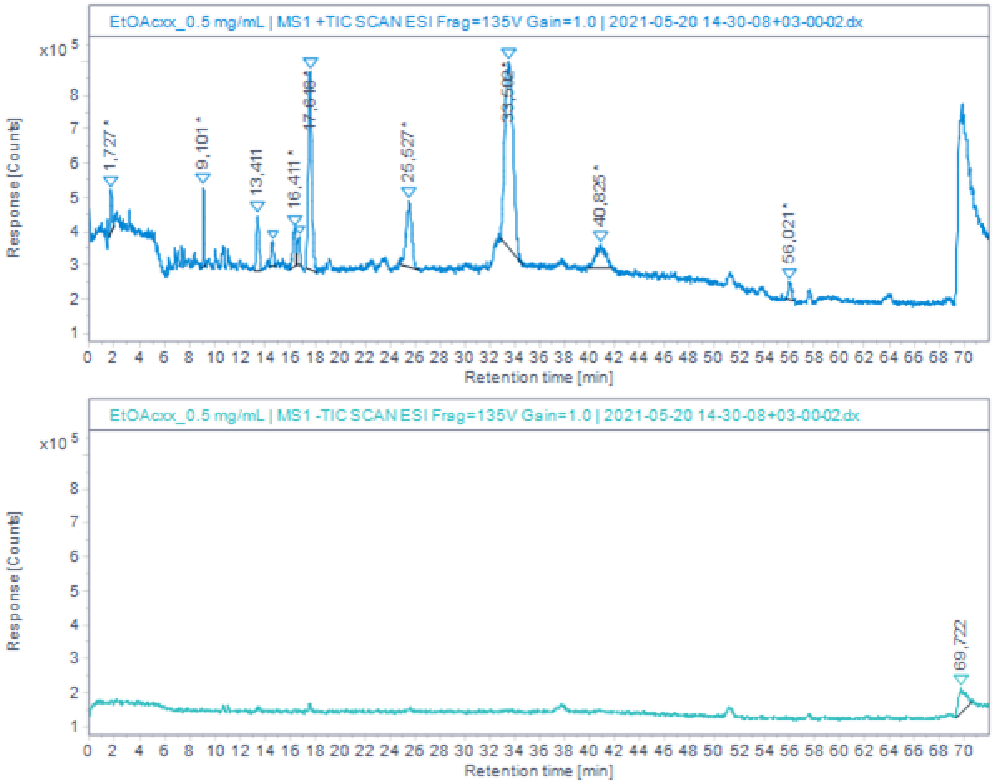

Figure S1

Figure S1. Total Ion chromatograph after positive (upper) and negative (lower) ionization of the SCP (EtOAc) extract.

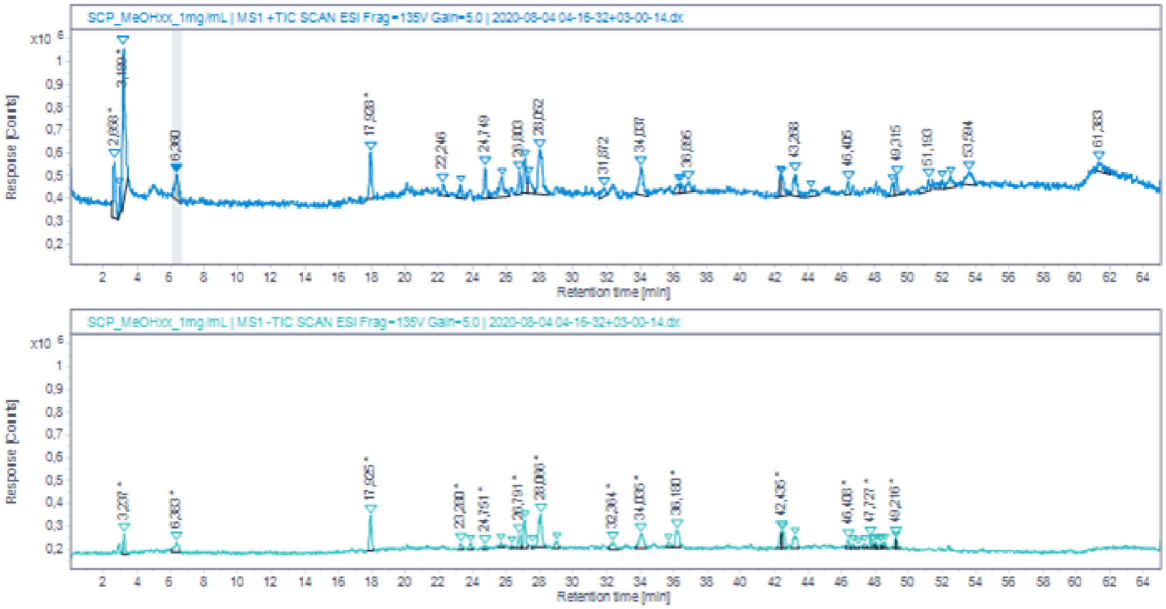

Figure S2

Figure S2. Total Ion chromatograph after positive (upper) and negative (lower) ionization of the SCP (MeOH) extract.

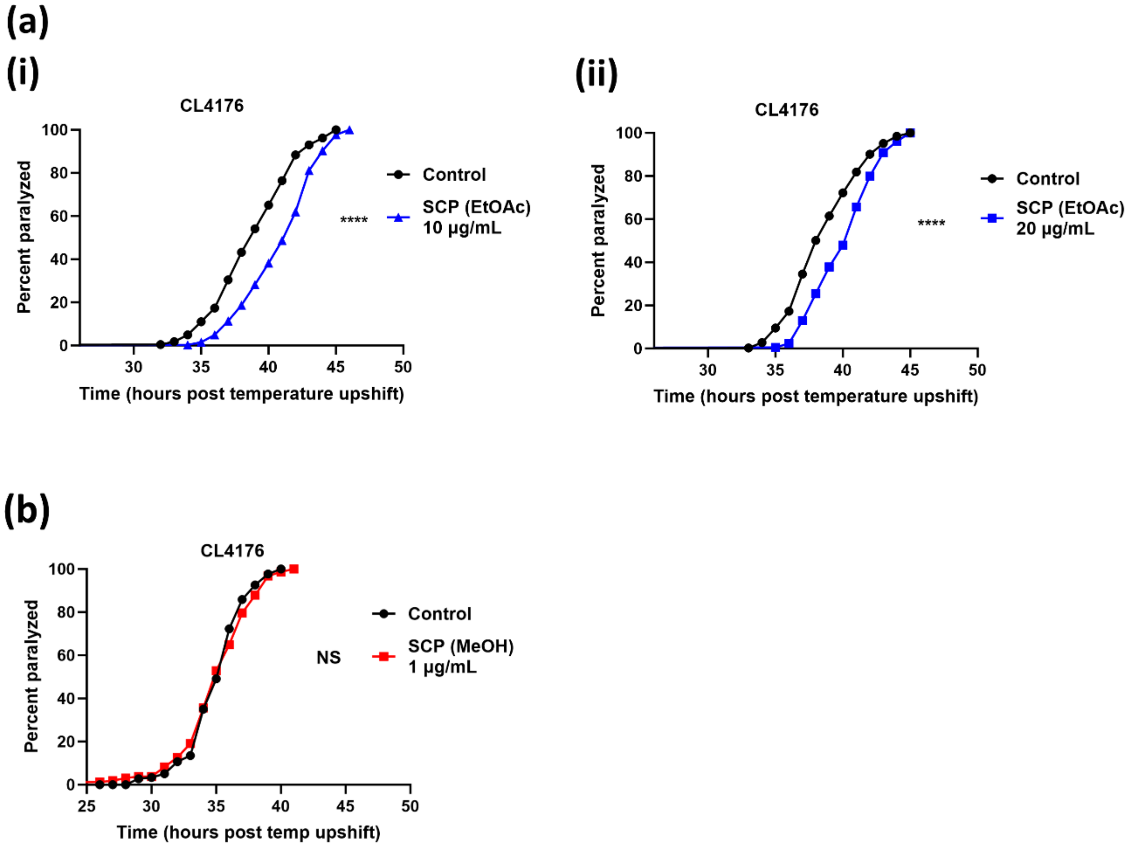

Figure S3

Figure S3. Treatment with SCP extracts confers protection against Aβ toxicity. Paralysis curves of CL4176 animals treated with SCP (**a**) EtOAc extract (10 µg/mL in (**i**) and 20 µg/mL in (**ii**)); (**b**) MeOH extract (1 µg/mL) or control (DMSO). (**a**) (**i**) Control: mean=39±0.63, n=1387, 10 µg/ml SCP (EtOAc) extract: mean=41±0.84, n=905, 5 independent experiments, ****P<0.0001; (**ii**) Control: mean=38.67±0.67, n=961, 20 µg/ml SCP (EtOAc) extract: mean=40.67±0.88, n=594, 3 independent experiments, ****P<0.0001; (**b**) Control: mean=36, n=177, 1 µg/ml SCP (MeOH) extract: mean=35, n=157, 1 independent experiment, NS (not significant). Median paralysis values are expressed as mean ± SEM. N denotes the number of animals that were paralyzed. Curves are the pooled result of the indicated independent experiments. For paralysis experiments the log-rank Mantel-Cox test was used.

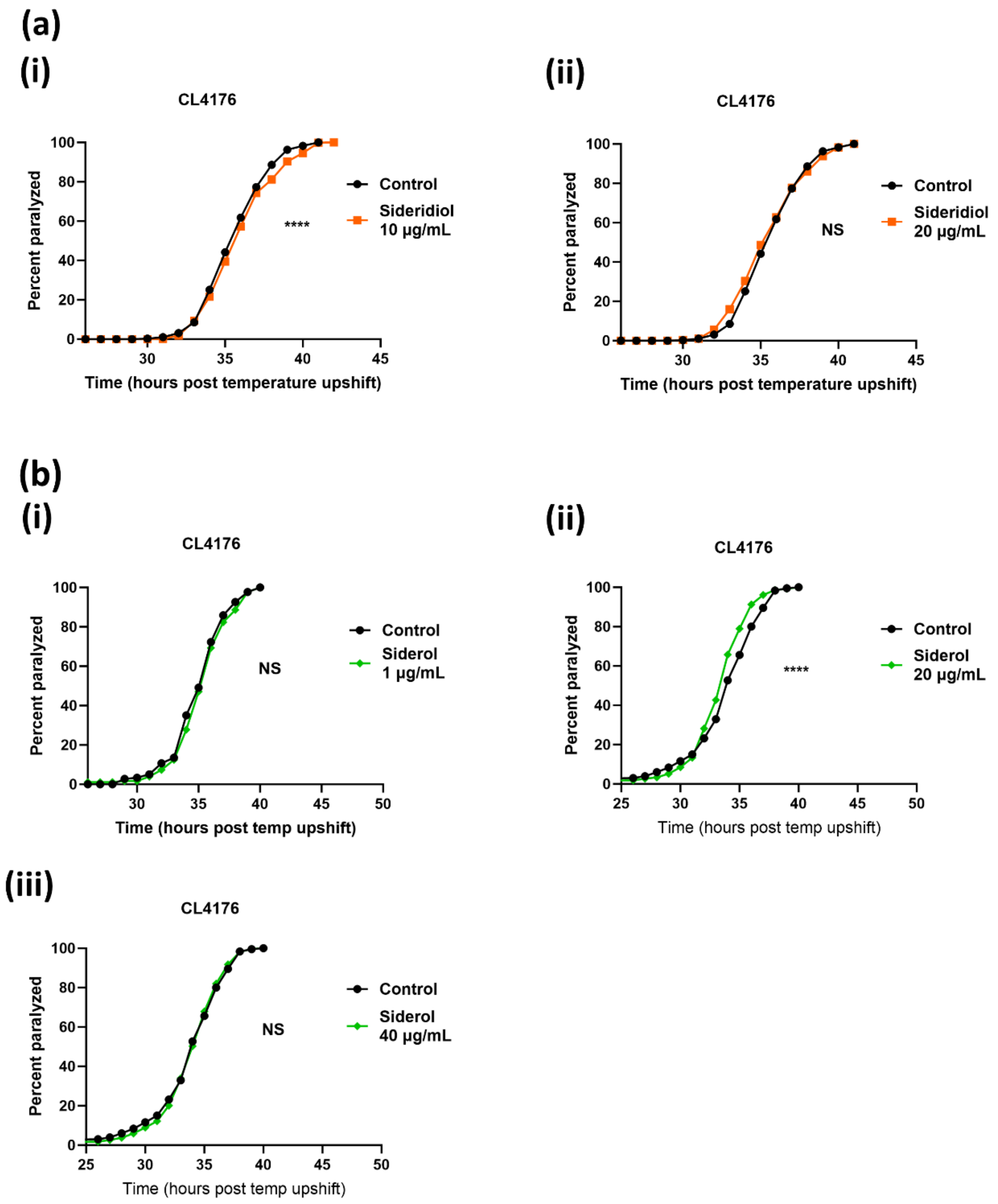

Figure S4

Figure S4. Sideridiol and not siderol is the main contributor of SCP (EtOAc) extract effects against Aβ toxicity. Paralysis curves of CL4176 animals treated with (**a**) (**i**) 10 µg/mL sideridiol; (**ii**) 20 µg/mL sideridiol; (**b**) (**i**) 1 µg/mL siderol; (**ii**) 20 µg/mL siderol; (**iii**) 40 µg/mL siderol or control (DMSO). (**a**) (**i**) Control: mean=36±0.58, n=1052, 10 µg/mL sideridiol: mean= 36±0.58, n=813, 3 independent experiments, ****P<0.0001; (**ii**) Control: mean=36±0.58, n=1052, 20 µg/mL sideridiol: mean= 36.33±0.88, n=866, 3 independent experiments, NS (not significant); (**b**) (**i**) Control: mean=36, n=177, 1 µg/mL siderol: mean=36, n=176, 1 experiment, NS (not significant); (**ii**) Control: mean=34.5±0.32, n=431, 20 µg/mL siderol:

mean=34±0, n=599, 2 independent experiments, ****P<0.0001; (**iii**) Control: mean=34.5±0.32, n=431, 40 μg/mL siderol: mean=34.5±0.41, n=501, 2 independent experiments, NS (not significant). Median paralysis values are expressed as mean ± SEM. N denotes the number of animals that were paralyzed. Curves are the pooled result of the indicated independent experiments. For paralysis experiments the log-rank Mantel-Cox test was used.

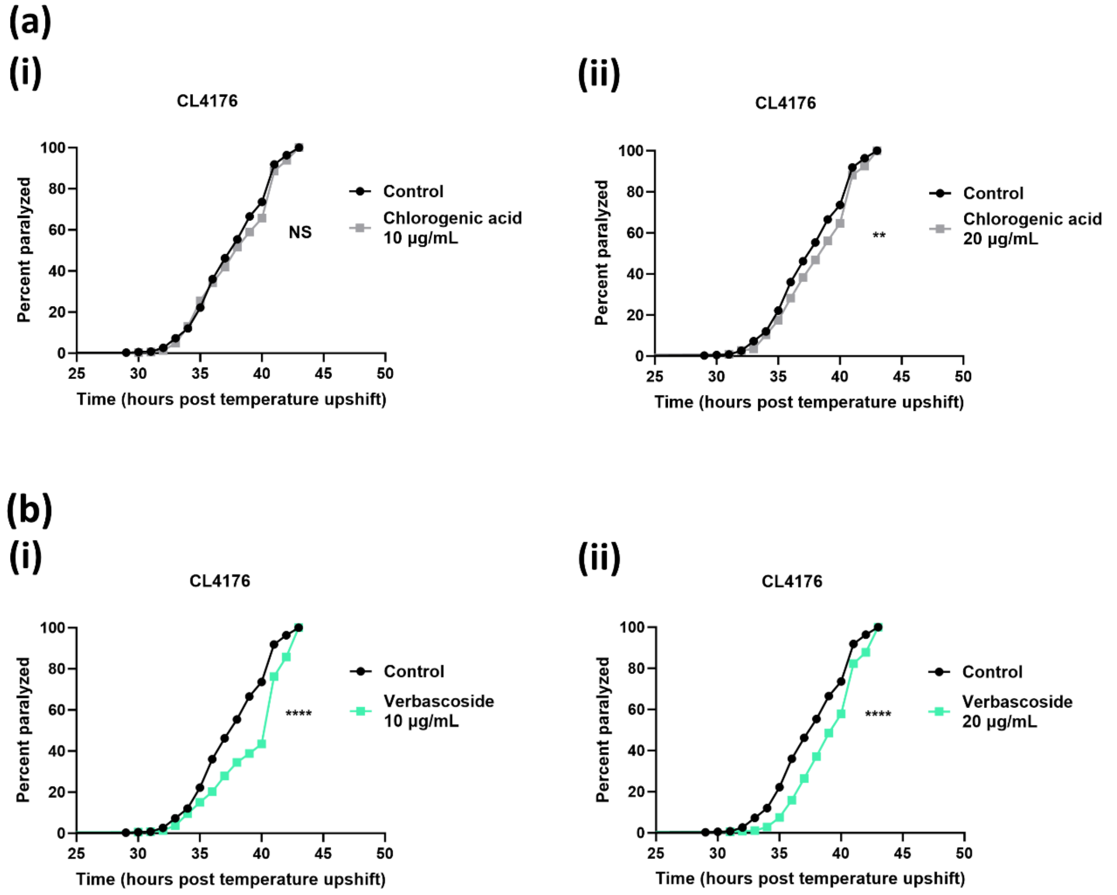

Figure S5

Figure S5. Verbascoside and not cholorogenic acid is the main contributor of SCP (MeOH) extract effects against Aβ toxicity. Paralysis curves of CL4176 animals treated with (**a**) (**i**) 10 μg/mL cholorogenic acid; (**ii**) 20 μg/mL cholorogenic acid and; (**b**) (**i**) 10 μg/mL verbascoside; and (**ii**) 20 μg/mL verbascoside or control (DMSO). (**a**) (**i**) Control: mean=38±1, n=383, 10 μg/mL cholorogenic acid: mean= 38.50±0.50, n=391, 2 independent experiments, NS; (**ii**) Control: mean=38±1, n=383, 20 μg/mL cholorogenic acid: mean= 39.50±1.50, n=337, 2 independent experiments, **P<0.01; (**b**) (**i**) Control: 38±1, n=383, 10 μg/mL verbascoside: mean=41±0, n=366, 2 independent experiments, ****P<0.0001; (**ii**) Control: 38±1, n=383, 20 μg/mL verbascoside: mean=39.50±0.50, n=344, 2 independent experiments, ****P<0.0001. Median paralysis values are expressed as mean ± SEM. N denotes the number of animals that were paralyzed. Curves are the pooled result of the indicated independent experiments. For paralysis experiments the log-rank Mantel-Cox test was used.
